# Comparative transcriptome analysis reveals that chlorophyll metabolism contributes to leaf color changes in wucai (*Brassica campestris* L.) in response to cold

**DOI:** 10.1186/s12870-021-03218-9

**Published:** 2021-09-28

**Authors:** Lingyun Yuan, Liting Zhang, Ying Wu, Yushan Zheng, Libing Nie, Shengnan Zhang, Tian Lan, Yang Zhao, Shidong Zhu, Jinfeng Hou, Guohu Chen, Xiaoyan Tang, Chenggang Wang

**Affiliations:** 1 College of Horticulture, Vegetable Genetics and Breeding Laboratory, Anhui Agricultural University, 130 West Changjiang Road, Hefei, 230036 Anhui China; 2 Provincial Engineering Laboratory for Horticultural Crop Breeding of Anhui, 130 West of Changjiang Road, Hefei, 230036 Anhui China; 3 Wanjiang Vegetable Industrial Technology Institute, Maanshan, 238200 Anhui China

**Keywords:** Wucai, Leaf color, Cold response, Transcriptome analysis, Chlorophyll biosynthesis, Carotenoid metabolism, Photosynthesis, Circadian rhythm

## Abstract

**Background:**

Chlorophyll (Chl) is a vital photosynthetic pigment involved in capturing light energy and energy conversion. In this study, the color conversion of inner-leaves from green to yellow in the new wucai (*Brassica campestris* L.) cultivar W7–2 was detected under low temperature. The W7–2 displayed a normal green leaf phenotype at the seedling stage, but the inner leaves gradually turned yellow when the temperature was decreased to 10 °C/2 °C (day/night), This study facilitates us to understand the physiological and molecular mechanisms underlying leaf color changes in response to low temperature.

**Results:**

A comparative leaf transcriptome analysis of W7–2 under low temperature treatment was performed on three stages (before, during and after leaf color change) with leaves that did not change color under normal temperature at the same period as a control. A total of 67,826 differentially expressed genes (DEGs) were identified. Kyoto Encyclopedia of Genes and Genomes (KEGG) pathway and Gene Ontology (GO) analysis revealed that the DEGs were mainly enriched in porphyrin and Chl metabolism, carotenoids metabolism, photosynthesis, and circadian rhythm. In the porphyrin and chlorophyll metabolic pathways, the expression of several genes was reduced [i.e. *magnesium chelatase subunit H* (*CHLH*)] under low temperature. Almost all genes [i.e. *phytoene synthase* (*PSY*)] in the carotenoids (Car) biosynthesis pathway were downregulated under low temperature. The genes associated with photosynthesis [i.e. *photosystem II oxygen-evolving enhancer protein 1* (*PsbO*)] were also downregulated under LT. Our study also showed that *elongated hypocotyl5* (*HY5*), which participates in circadian rhythm, and the metabolism of Chl and Car, is responsible for the regulation of leaf color change and cold tolerance in W7–2.

**Conclusions:**

The color of inner-leaves was changed from green to yellow under low temperature in temperature-sensitive mutant W7–2. Physiological, biochemical and transcriptomic studies showed that HY5 transcription factor and the downstream genes such as *CHLH* and *PSY*, which regulate the accumulation of different pigments, are required for the modulation of leaf color change in wucai under low temperature.

**Supplementary Information:**

The online version contains supplementary material available at 10.1186/s12870-021-03218-9.

## Background

Color vegetable is a kind of vegetable with special color in some edible organs, which is different from common varieties. The formation of leaf color traits of color vegetables is a very complex metabolic process and is primarily determined by the lipid-soluble pigments in leaves. In most plants, leaf color is determined by the retention or production of three main types of pigments: chlorophylls, carotenoids, and anthocyanin [1, 2]. Carotenoids are accessory pigments that transfer absorbed light to chlorophylls [3]. The Chl molecules, which harvests light energy and drives electron transfer in the reaction center (RC), are universal in photosynthetic organisms [4, 5]. The amount of Chl content and the morphology and structure of chloroplast largely determine the photosynthetic efficiency of plants [6]. The porphyrin and Chl metabolism pathways consist of three common steps: Chl synthesis, Chl cycle, and Chl degradation [7]. Chl synthesis is catalyzed by 17 enzymes [8], among which, Mg-chelatase is a key enzyme in Chl biosynthesis, which catalyzes the formation of Mg porphyrin IX from Mg^2+^ and protoporphyrin IX in plants. Mg-chelatase is composed of four components, the H subunit (CHLH/ABAR), I subunit (CHLI), D subunit (CHLD), and GENOMES UNCOUPLED 4 (GUN4) protein, which jointly regulate the activity of Mg-chelatase in Chl biosynthesis [9]. In Chl biosynthesis pathway, glutamic acid (Glu), 5-aminolevulinic acid (ALA), procyanidin (PBG), protoporphyrin IX, Mg-protoporphyrin IX, and protochlorophyllide (Pchlid) are the intermediate metabolites of Chl. Chl can be degraded via a catabolic pathway, leading to a decrease of colored pigments and accumulation of colorless metabolic products, which in turn drives the color change in leaves [10, 11]. When the Chl is degraded due to aging or stress, leaves are gradually changed to yellow, the color of which represents the content of Car.

Many temperature-sensitive mutants exhibit different leaf colors at different temperatures, and most of them grow poorly or even die after discoloration. A rice Chl-deficient (*tcd3*) mutant displays an albino phenotype before the four-leaf stage and ultimately dies when grown at 20 °C, but grows normally at 32 °C [12]. The rice *tcd12* mutant displays albino leaves, chloroplast deformity, and decreased Chl contents at low temperatures [13]. At 22 °C, 26 °C, and 29 °C, the leaves of Arabidopsis mutant *tsc1* are green, light green, and white respectively [14]. In tomato, mutation in *WV* gene leads to a reduced Chl content in the young leaves under low temperature and high light intensity conditions [15]. The inner leaves of a novel wucai cultivar W7–2 exhibit green color at the early leaf stage under the temperature of 24–33 °C, but they turn yellow under the temperatures of 8–18 °C. Although the inner leaves turn yellow, the plants grow normally in the winter, indicating that the change in leaf color does not affect the whole growth cycle [16].

Wucai (*Brassica campestris* L*. ssp. chinensis var. rosularis* Tsen) is a subspecies of Chinese cabbage (*B. campestris* L*.*). It is a nutritious vegetable enriched with minerals and vitamins, and it exhibits colorful leaves. The diversity of leaf color caters to the demand of consumers in various degrees, and the appearance quality of wucai is greatly improved after the change of the leaf color. Therefore, exploring the regulation mechanisms of wucai leaf color formation will provide valuable information for breeding and cultivating the varieties of wucai with excellent colors that are favored by consumers. So far, only a few studies have reported the phenomenon of the color change in multicolored leaves, and the underlying mechanisms remain unclear [12–15, 17]. In wucai cultivar W7–2, the leaves at the adult stage turn yellow when the temperature gradually declines in the winter [16]. In the present study, stable genetic descendants of W7–2 were selected as the study materials, and the molecular mechanism of leaf color change under low temperature was investigated through comprehensive biochemical, bioinformatic, and transcriptomic analyses.

## Results

### Analysis of leaf color parameter changes at the adult stage

**Fig. 1:**
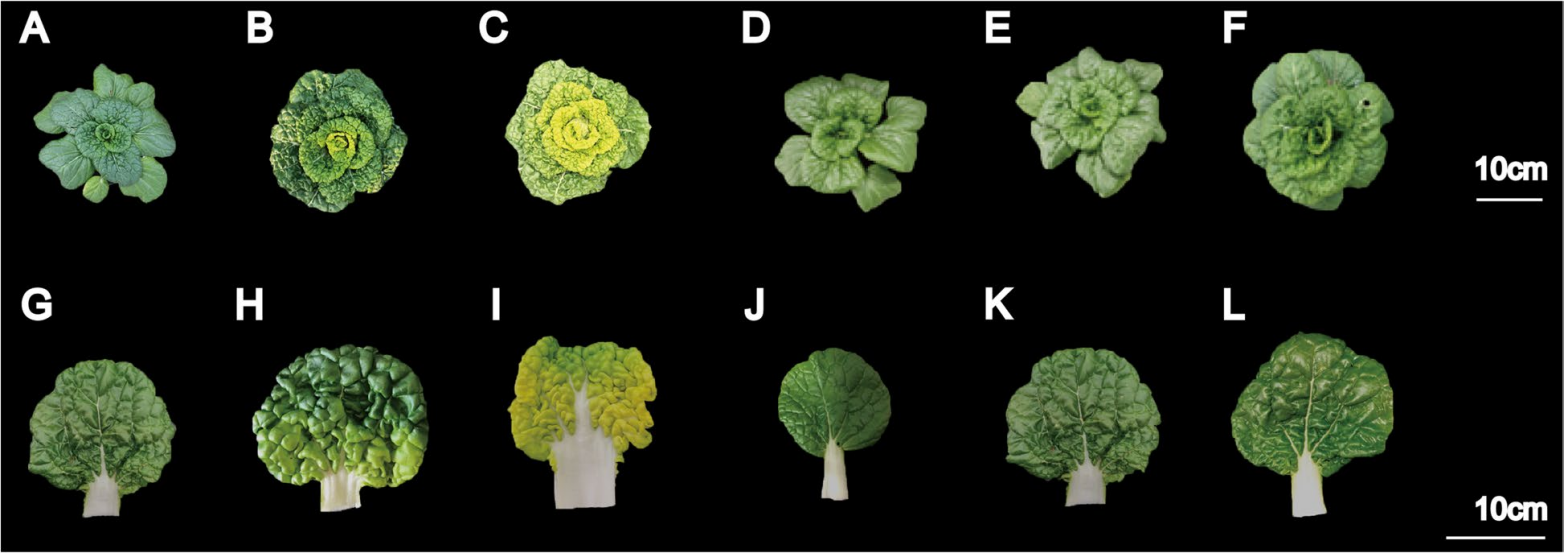
Phenotypes of individual W7–2 plants and leaves under LT (**A**–**C**, **G**–**I**) and NT (**D**–**F**, **J**–**L**) treatments. LT, low temperature; NT, normal temperature

**Fig. 2:**
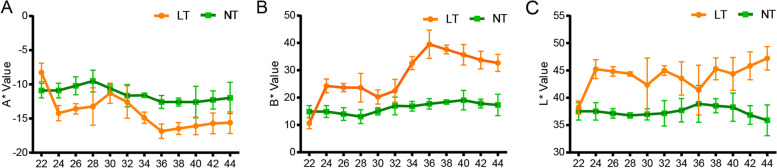
Changes in color A* (**A**), B* (**B**), and L* (**C**) of the two Wucai varieties at different DAPs. LT, low temperature; NT, normal temperature. Error bars represent ± SD. Data were derived from five independent experiments

W7–2 leaves displayed a green color at both seedling and adult stages under normal temperature (NT). Under low temperature (LT) conditions, however, W7–2 cultivar exhibited green-leaf phenotype at the seedling stage, but its inner green leaves gradually turned yellow at the adult stage under LT (Fig. 1). The leaf color parameters (value L*, value a*, and value b*) varied at different growth and development stages. Compared to NT, value b* (yellowness and blueness) remarkably increased over time under LT (Fig. 2B). Especially, at the stages of 30–36 DAP (day after planting), the value b* increased from 20.24 to 39.52. Value a* (redness and greenness) was not significantly changed under NT but slightly decreased in LT (Fig. 2A). No significant differences were detected for Value L* (lightness) under both NT and LT (Fig. 2C). At 24 DAP, group LTB (low temperature before color change) were selected for green leaves under LT, and group NTB (normal temperature before color change) were green leaves under NT. At 34 DAP, half-yellow and half green-leaf samples under LT were referred to as LTC (low temperature during color change), and green-leaf samples under NT were referred to as NTC (normal temperature during color change). At 44 DAP, yellow leaves under LT were referred to as LTA (low temperature after color change), and green leaves under NT were referred to as NTA (normal temperature after color change).

### Measurement of pigment contents

**Fig. 3:**
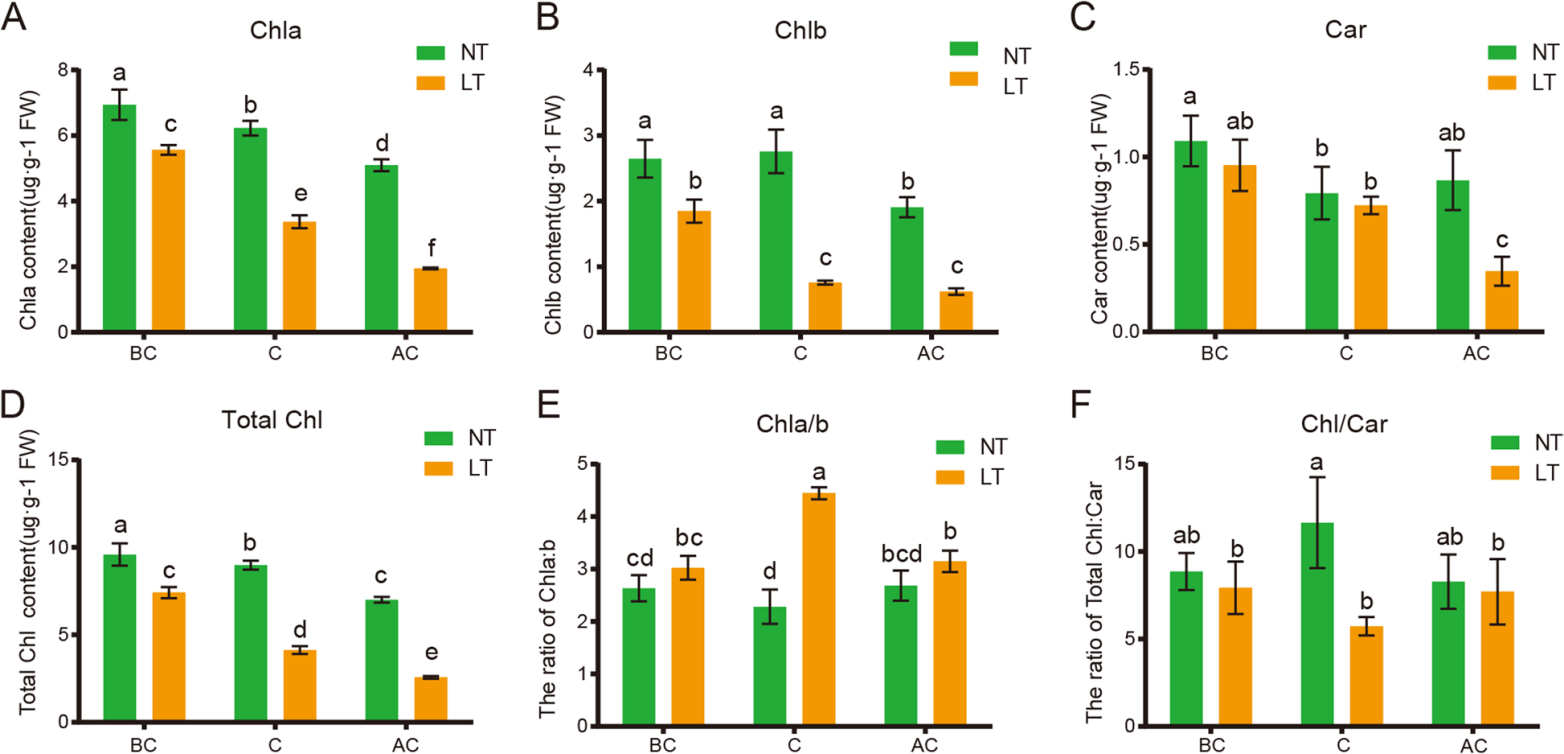
Comparison of pigment (Chl and Car) contents under LT and NT at different periods after transplanting. The contents of Chl a (**A**), Chl b (**B**), total Chl (**C**), Car (**D**), the Chl a/b ratio (**E**), and the total Chl/Car ratio (**F**) in the leaves under NT and LT were measured. LT, low temperature; NT, normal temperature; BC, before color change; C, color change; AC, after color change. Data were derived from three biological replicates of each cultivar. Pigments were measured three times. Error bars represent ± SD. Different letters indicate significant differences (*p* < 0.05)

To understand the low temperature-induced color change in W7–2, the Chl and Car contents in plant leaves were measured under both NT and LT. Our result showed that the contents of Chl a, Chl b, total Chl and Car were decreased under NT, but they were more dramatically reduced under LT (*p* < 0.05) (Fig. 3A, B, C, D). The Chl a/b ratio was higher under LT than that under NT at later growth stages, while the total Chl/Car ratio was higher under NT (Fig. 3E, F).

### Measurement of Chl intermediate metabolites

**Fig. 4:**
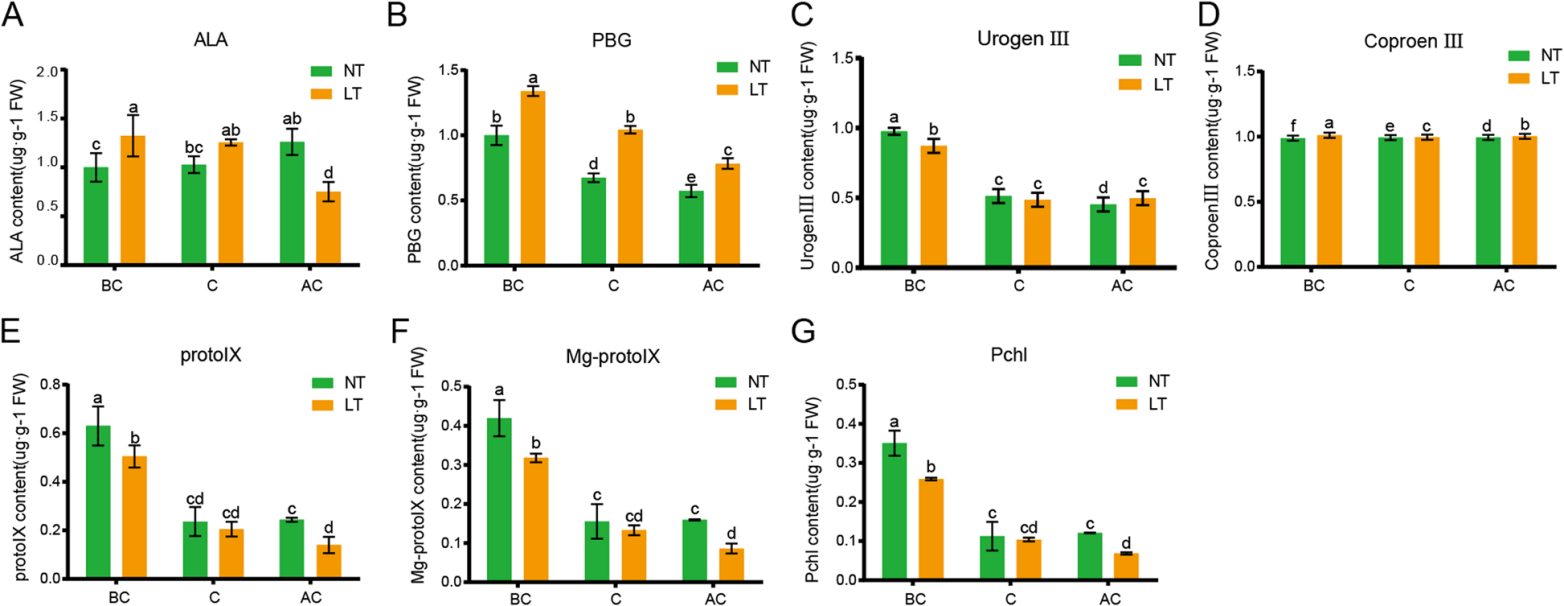
The concentrations of chlorophyll synthesis intermediates at different DAP in the two varieties under LT and NT. The concentrations of δ-aminolevulinic acid (**A**), porphobilinogen (**B**), uroporphyrinogen III (**C**), coproporphyrin III (**D**), protoporphyrin IX (**E**), Mg-protoporphyrin IX (**F**), and protochlorophyllide (**G**) were measured. LT, low temperature; NT, normal temperature; BC, before color change; C, color change; AC, after color change. Data were quantified using three biological replicates of each cultivar. Each data point represents the mean (± SD) of three separate experiments. Different letters within a column indicate significant differences (p < 0.05)

The contents of Chl metabolic intermediates were measured under LT and NT. The results revealed that ALA was increased slightly under NT, but decreased significantly under LT, especially from LTC to LTA. The ALA content in complete yellowed leaves was lower under LT than that under NT. The PBG content was decreased under both NT and LT, and was lower at NT than LT. The content of protoporphyrin IX was decreased significantly under NT and LT, especially from LTB to LTA. From LTC to LTA, only the protoporphyrin IX content was significantly decreased under NT and was higher under LT than that under NT. Interestingly, the contents of protoporphyrin IX, Mg-proto IX, and Pchlide exhibited a same trend, and the contents of Chl intermediate metabolites decreased significantly as plant growth progressed (Fig. 4).

### Effects of low temperature on Chl fluorescence

**Fig. 5:**
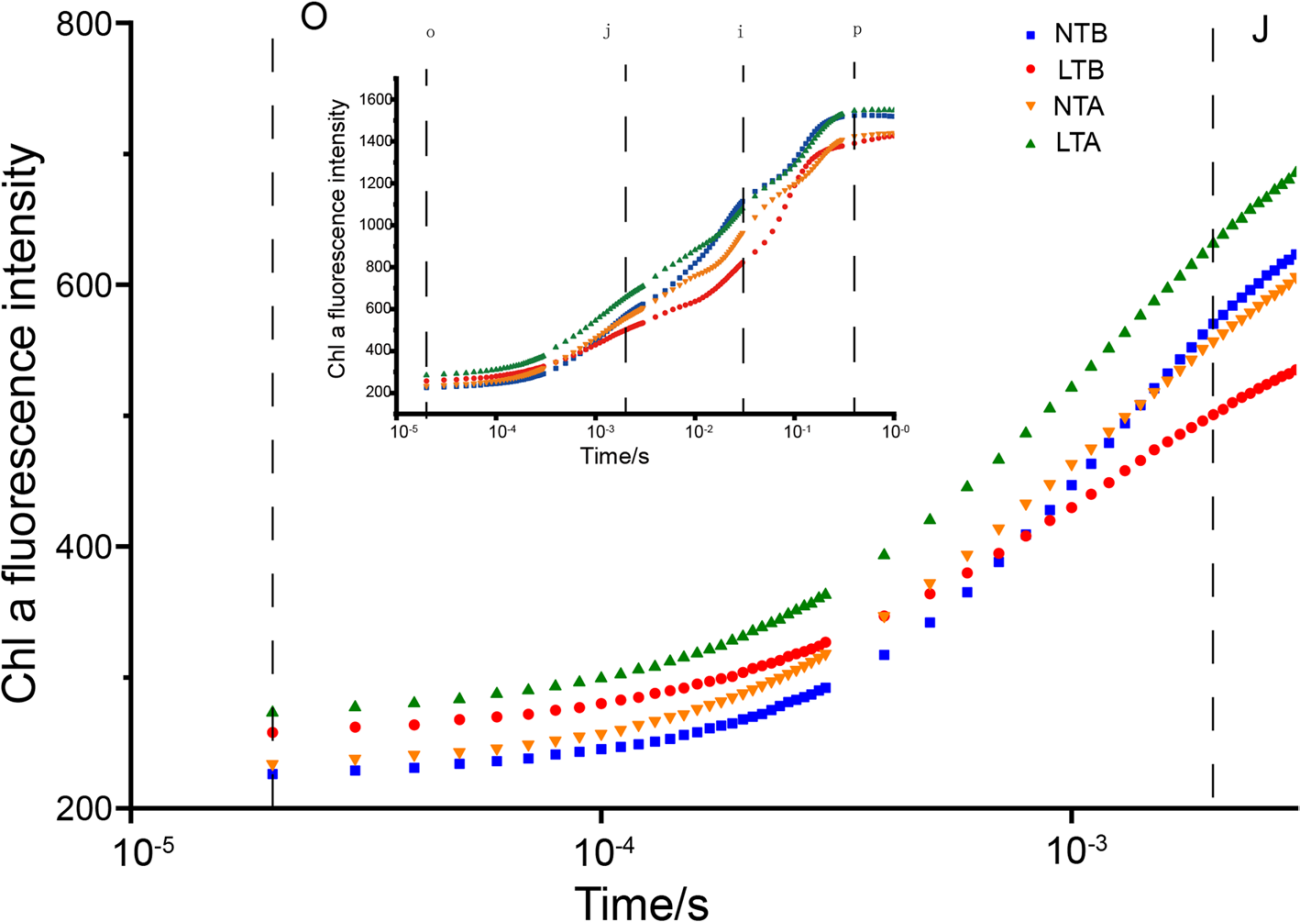
The fast chlorophyll a fluorescence transient (OJIP) under NT and LT is plotted on a logarithmic time scale (0.00001–1 s). LTA, low temperature after color change; LTB, low temperature before color change; NTA, normal temperature after color change; NTB, normal temperature before color change. Transient curves of each line represent the average of five measurements per treatment

**Fig. 6:**
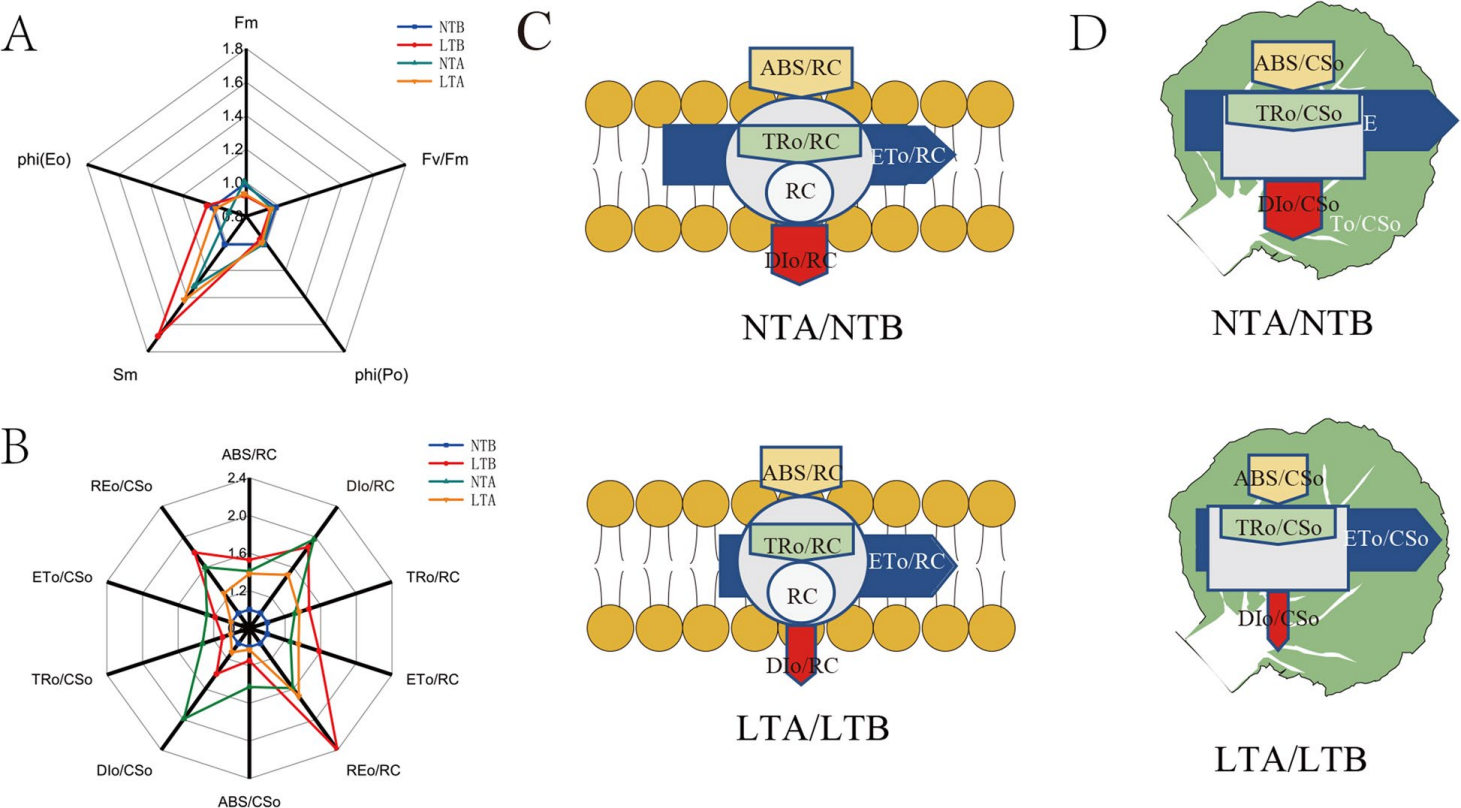
Radar plot showing various technical fluorescence parameters (**A**, **B**). Each line represents the average of five measurements per treatment. Energy pipeline models of specific energy fluxes (membrane model) (**C**) and phenomenological energy fluxes (leaf model) (**D**) under LT and NT. LTA, low temperature after color change; LTB, low temperature before color change; NTA, normal temperature after color change; NTB, normal temperature before color change; Fm, maximum fluorescence; Fv, variable fluorescence; φPo, maximum quantum yield for primary photochemistry; Sm, normalised total complementary area above the O-J-I-P transie; φEo, quantum yield for electron transport; RC, reaction centre; RC/CS, the concentration of active PSII reaction centres per excited cross section; ABS/RC, absorption of flux per RC; DIo/RC, Dissipated energy flux per RC; TRo/RC, Trapped energy flux per RC; ETo/RC, Electron transport flux per RC; ABS/DIo, absorption/ dissipated energy; ABS/CSo, Absorption of flux per CS; DIo/CSo, Dissipated energy flux per CS; TRo/CSo, Trapped energy flux per CS; ETo/CSo, Electron transport flux per CS

Chl fluorescence transients, which typically consist of O, J, I, and P phases, were measured under different treatments (Fig. 5A). The effects of stress on the active RCs of plants were determined by JIP-assays. The fluorescence of the main quinone receptor of electron Q_A_ in PSII (O-J phase) was higher at NTA than that under NTB, and thus the donor side activity of PSII was enhanced under NT. However, there were no significant differences in fluorescence between LTA and LTB, indicating that LT neither damaged nor enhanced the activity of the PSII donor side. The level of point I mainly depends on the heterogeneity of the plastoquinone (PQ) pool. In the characteristic activities of fluorescence quenching and the water-splitting system (J-I phase), as controlled by the PSII donor site, NTB and NTA were similar and higher than LTA, while LTB was the lowest. Additionally, leaves under NT had a weaker P peak. Fm (maximum fluorescence), Fv/Fm (variable fluorescence / maximum fluorescence) and φPo (maximum quantum yield for primary photochemistry) were the highest in group NTB, followed by NTA, LTB and LTA (Fig. 6A). Sm (normalised total complementary area above the O-J-I-P transie) rose to 131% after NT treatment, while it was decreased to 84.03% after LT treatment (Fig. 6A). φEo (quantum yield for electron transport) decreases slightly at NT and LT (Fig. 6A). Compared to NTB, ABS/RC (absorption of flux per RC), DIo/RC (dissipated energy flux per RC), TRo/RC (trapped energy flux per RC), ETo/RC (Electron transport flux per RC), and REo/RC (electron transfer per RC to the end of PSI) in NTA were increased by 1.40, 1.96, 1.31, 1.26, and 1.58 times, respectively, while ABS/DIo (absorption/ dissipated energy) in NTA dropped to 71% of NTB. Compared to LTB, the ABS/RC, DIo/RC, TRo/RC, ETo/RC, and REo/RC in LTA were increased by 0.90, 0.80, 0.93, 0.85, and 0.71 times, respectively, while the ABS/DIo increased was by 1.12 times. Compared to NTB, ABS/CSo (absorption of flux per CS), DIo/CSo (dissipated energy flux per CS), TRo/CSo (trapped energy flux per CS), ETo/CSo (electron transport flux per CS), and REo/CSo (electron transfer per CS to the end of PSI) in NTA were increased by 1.42, 1.99, 1.33, 1.28, and 1.60 times respectively, while ABS/DIo was decreased to 71% of NTB. Compared to LTB, ABS/CSo, DIo/CSo, TRo/CSo, ETo/CSo, and REo/CSo in LTA were 0.90, 0.80, 0.91, 0.85, and 0.70 times of that in LTB, respectively, while ABS/DIo was increased by 1.12 times compared with LTA. ABS/CS and DIo/CS were decreased in LT, and the absorption of energy satisfied the energy requirements under LT. However, ABS/CS and DIo/CS were increased under NT, and the ratio of energy absorption and consumption was < 1, indicating the absorbed energy under NT did not meet the energy requirements (Fig. 6).

### Mapping and quantitative assessment of Illumina sequences

In order to understand the molecular basis of wucai leaf color changes, the DEGs between LT and NT were analyzed using the leaves of LTA, LTB, LTC, NTA, NTB, and NTC. An Illumina Seq platform was used to sequence the six samples with three biological replicates. Six libraries were constructed for high-throughput sequencing. To identify the DEGs, we obtained a total of 931.64 MB raw reads and 139.76 GB raw bases. After filtering, 914.75 MB clean reads and 128.53 GB clean bases were obtained. The Illumina qphredQ30 of all samples was > 90% (Additional file 1 Table S1).

**Fig. 7:**
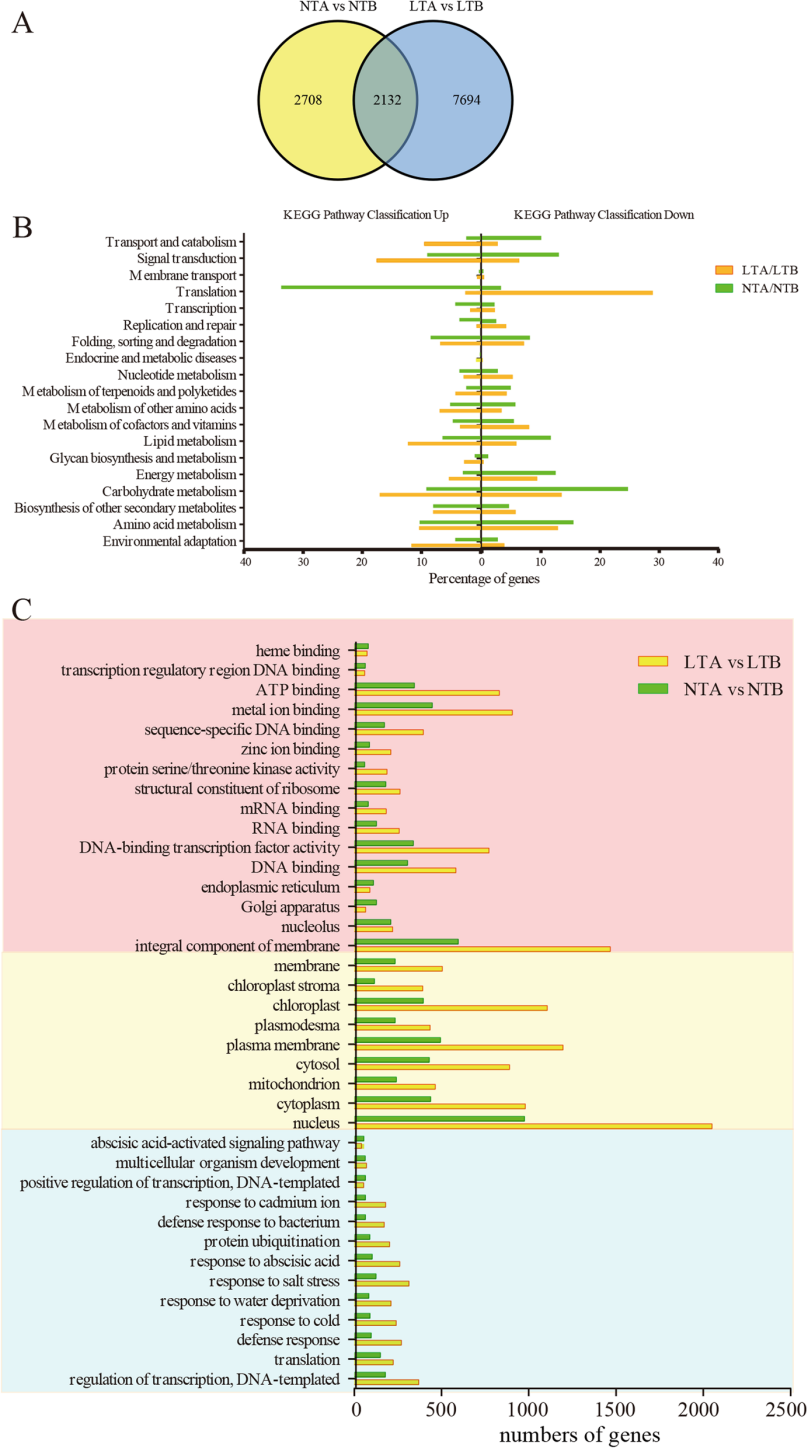
Functional annotations of the unigenes obtained from W7–2 leaf transcriptomes. **A** Venn diagram of differentially expressed genes (DEGs) under NT and LT. **B** Analysis of DEGs based on KEGG pathway annotations of W7–2 transcripts in LTA/LTB and NTA/NTB. LTA, low temperature after color change; LTB, low temperature before color change; NTA, normal temperature after color change; NTB, normal temperature before color change. The vertical axis shows the annotations of the KEGG metabolic pathways, and the horizontal axis represents the percentage of genes. The left genes were upregulated, and the right genes were downregulated. **C** GO analysis of the DEGs. The vertical axis shows the annotations of the GO metabolic pathways, and the horizontal axis represents the numbers of genes

Based on the criteria of *p* < 0.05 and |log_2_FC| > 1, 9825 DEGs (5260 upregulated and 4565 downregulated) were identified by comparing LTA and LTB, and 4839 DEGs (3211 upregulated and 1628 downregulated) were identified by comparing NTA and NTB (Fig. 7A). By comparing these two sets of DEGs, 7694 unique genes were found in LTA/LTB (with LTB as the control, DEGs were screened in LTA) set. These unique genes included those that show altered gene expression after LT treatment, which facilitates us to mine the genes responsible for leaf color change.

### Gene functional annotation and classification

The GO functional enrichment analysis of the DEGs was performed in NTA/NTB (with NTB as the control, DEGs were screened in NTA) and LTA/LTB set. Transcriptional regulation (GO:0006355), defense response (GO:0006952), and response to cold (GO:0009409), were ranked the highest among all GO biological processes between LT and NT. Based on cellular component, the DEGs were enriched in the nucleus (GO:0005634), cytosol (GO:0005829), chloroplast (GO:0009507), and some membrane pathways (GO:0005886, GO:0009506, GO:0009570, GO:0016020, and GO:0016021). Based on molecular function, the DEGs were enriched in DNA binding (GO:0003677), DNA-binding transcription factor activity (GO:0003700), RNA binding (GO:0003723), and structural constituent of ribosome (GO:0003735) (Fig. 7C).

Furthermore, we analyzed the KEGG pathways of enriched DEGs. Translation accounted for 28.91% of all downregulated genes in LTA/LTB, ranking the first in all KEGG pathways, but only accounted for 2.58% of all upregulated genes in LTA/LTB. 33.72% of upregulated genes and 3.26% of downregulated genes in NTA/NTB set belonged to translation pathway. 17.56% upregulated and 6.35% downregulated gene in LTA/LTB set, and 9.04% upregulated and 13.04% downregulated gene in NTA/NTB set belonged to signal transduction pathway. For the energy metabolism pathway, 17.06% of genes were up-regulated and 9.37% of genes were downregulated in LTA/LTB set, and increased by 3.01% of genes were up-regulated and 12.5% of genes were downregulated in NTA/NTB set. 11.71% of upregulated gene in LTA/LTB set, and less than 5% of downregulated genes under NT belonged to environmental adaptation pathway. The DEGs in LTA/LTB and NTA/NTB were also enriched in folding, sorting and degradation, nuclear metabolism, metabolism of terpenoids and polyketides, and metabolism of other amino acids (Fig. 7B).

### DEGs of the porphyrin and Chl metabolism pathway

**Fig. 8:**
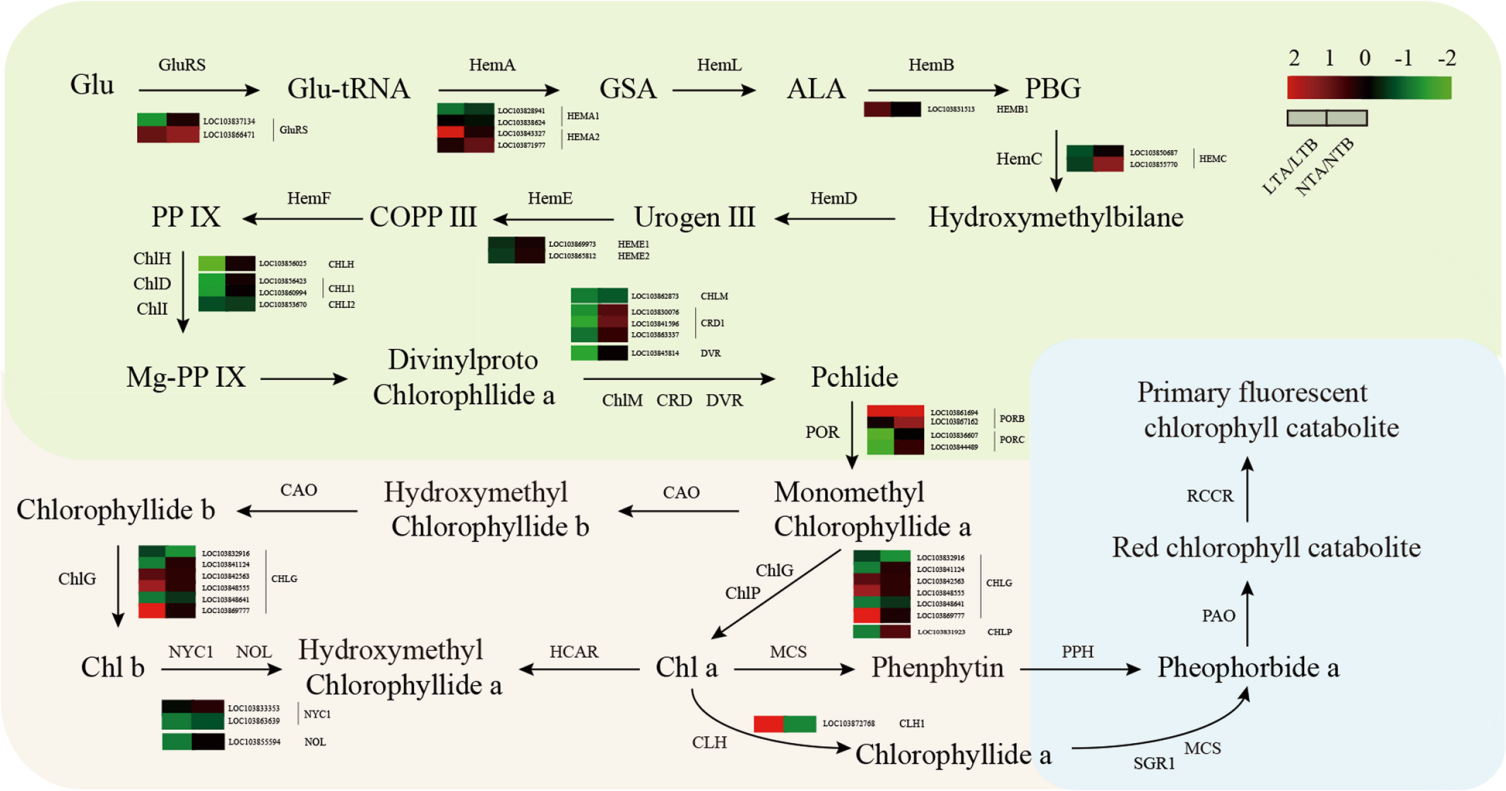
Heatmap of DEGs related to porphyrin and chlorophyll metabolism, and the measurement of chlorophyll intermediate metabolites. LTA, low temperature after color change; LTB, low temperature before color change; NTA, normal temperature after color change; NTB, normal temperature before color change; LTA/LTB, with LTB as the control, DEGs were screened in LTA; NTA/NTB, with NTB as the control, DEGs were screened in NTA; GluRS, *Glutamyl/glutaminyl-tRNA synthetase*; *HEMA*, *glutamyl-tRNA reductase*; *HEML*, *glutamate-1-semialdehyde 2,1-aminomutase*; *HEMB*, *porphobilinogen synthase*; *HEMC*, *hydroxymethylbilane synthase*; *HEMD*, *uroporphyrinogen-III synthase*; *HEME*, *uroporphyrinogen decarboxylase*; *HEMF*, *coproporphyrinogen III oxidase*; *CHLH*, *magnesium chelatase subunit H*; *CHLM*, *Magnesium-protoporphyrin IX methyltransferase*; *DVR*, *3,8-Divinyl protochlorophyllide a 8-vinyl reductase*; *CRD*, *Chloroplast Relocation Defective*; *POR*, *NADPH-protochlorophyllide oxidoreductase*; *CAO*, *chlorophyllide a oxygenase*; *CHLG*, *chlorophyll/bacteriochlorophyll a synthase*; *CLH*, *chlorophyllase*; *CHLP*, *geranylgeranyl reductase*; *HCAR*, *7-hydroxymethyl chlorophyll a reductase*; *NOL*, *non-yellow coloring-like*; *NYC1*, *non-yellow coloring*; *SGRL*, *Stay-Green Rice like*; *PaO*, *pheophorbide a oxygenase*; *RCCR*, *red chlorophyll catabolite reductase*

Chl metabolism includes Chl biosynthesis, Chl cycle, and Chl degradation. Interruptions in these processes result in leaf color changes. Therefore, we carefully analyzed the expression of the genes encoding the enzymes involved in this metabolic pathway. A total of 91 candidate genes related to porphyrin and Chl metabolism pathways were identified and analyzed. After removing the genes that did not match the criteria of *p* < 0.05 and |log_2_FC| > 1, the remaining 37 DEGs were analyzed in this study (Additional file 4: Table S4). Among them, 28 DEGs were downregulated and six were up regulated under LT, and two DEGs were downregulated and seven were up regulated under NT (Fig. 8). The expression levels of *CHLH*, *CHLI*, *CHLD*, and *GUN4*, which encode the four key components of Mg-chelatase, were downregulated under LT, but they were not significantly changed under NT. Mg-chelatase is the key enzyme in the synthesis of protoporphyrin IX to Mg-protoporphyrin IX and plays an important role in Chl synthesis. Three DEGs *DVR*, *CRD*, and *CHLM*, which encode key enzymes that are involved in the transition from divinylproto chlorophyllide a to Pchlide, were also downregulated under LT, but not under NT. Among the DEGs involved in Chl cycle, *NOL* and *NYC1* were downregulated and *CLH* was upregulated under LT. Among the DEGs under both NT and LT, no genes were identified that are associated with Chl degradation.

### DEGs of the Car biosynthesis pathway

**Fig. 9:**
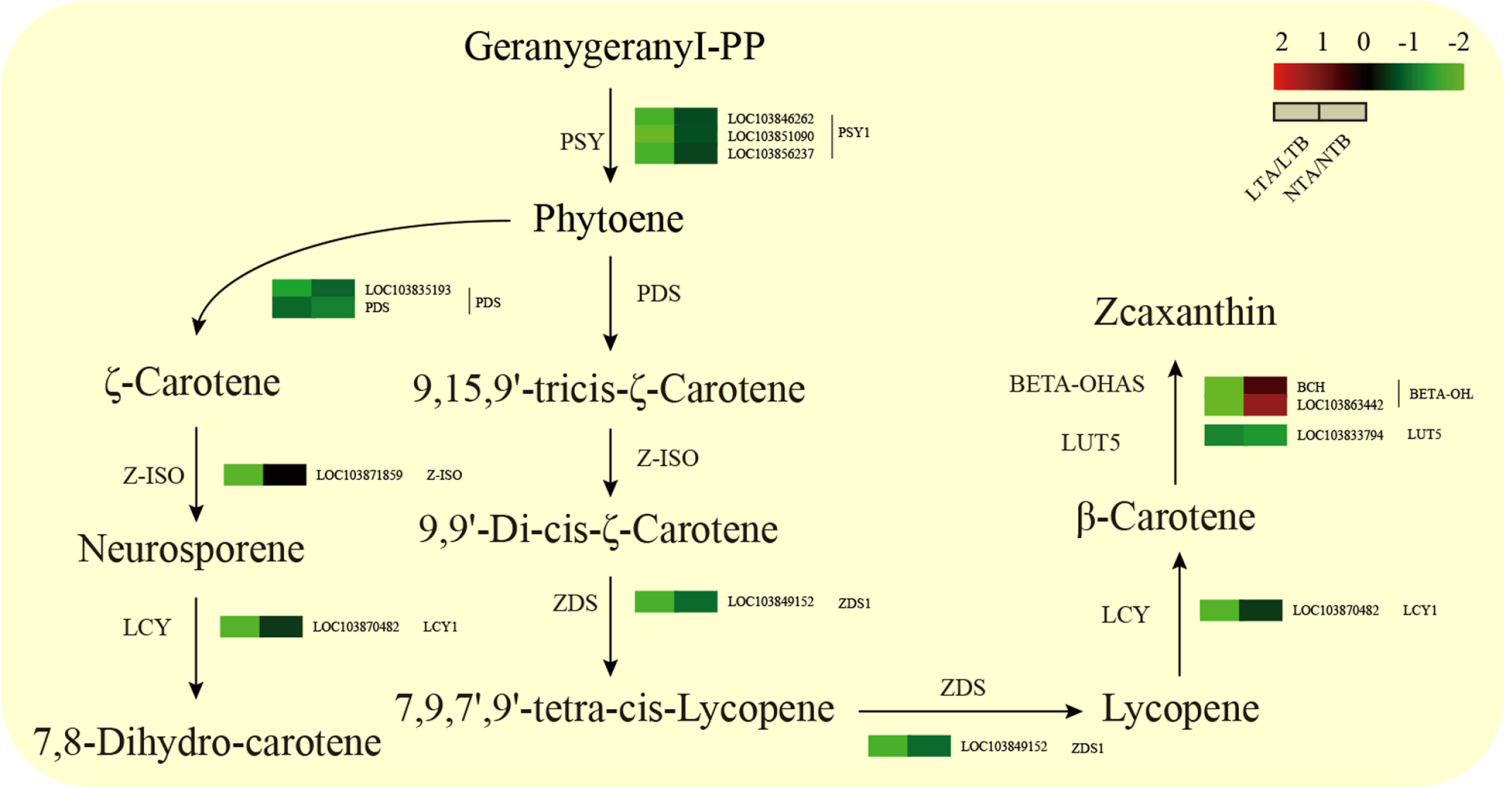
Heatmap of DEGs related to the carotenoids biosynthesis pathways. LTA, low temperature after color change; LTB, low temperature before color change; NTA, normal temperature after color change; NTB, normal temperature before color change; LTA/LTB, with LTB as the control, DEGs were screened in LTA; NTA/NTB, with NTB as the control, DEGs were screened in NTA; *PSY*, *phytoene synthase*; *PDS*, *phytoene desaturase*; *Z-ISO, zeta-carotene isomerase*; *ZDS*, *ζ- carotene desaturase*; *CRTISO*, *Carotene Cis-Trans Isomerase*; *LCY*, *lycopene beta cyclas*e; *LUT5*, *enhanced five-input lookup table*; *BETA-OHASE*, *beta-carotene 3-hydroxylase 1*

Car refers to carotenoids and their oxidative derivatives (lutein). Phytoene synthase (PSY) and phytoene desaturas*e* (PDS) are two key enzymes involved in Car biosynthesis pathway. Interruption of these enzymatic reactions may lead to changes in leaf color. Our transcriptome data showed that almost all genes in the Car biosynthesis pathway including *PSY*, *PDS*, *ζ- carotene desaturase* (*ZDS*), *zeta-carotene isomerase* (*Z-ISO*), and *lycopene beta cyclase* (*LCY*) were downregulated under LT (Fig. 9, Additional file 5: Table S5).

### DEGs of photosynthesis and the photosynthesis-antenna pathway

**Fig. 10:**
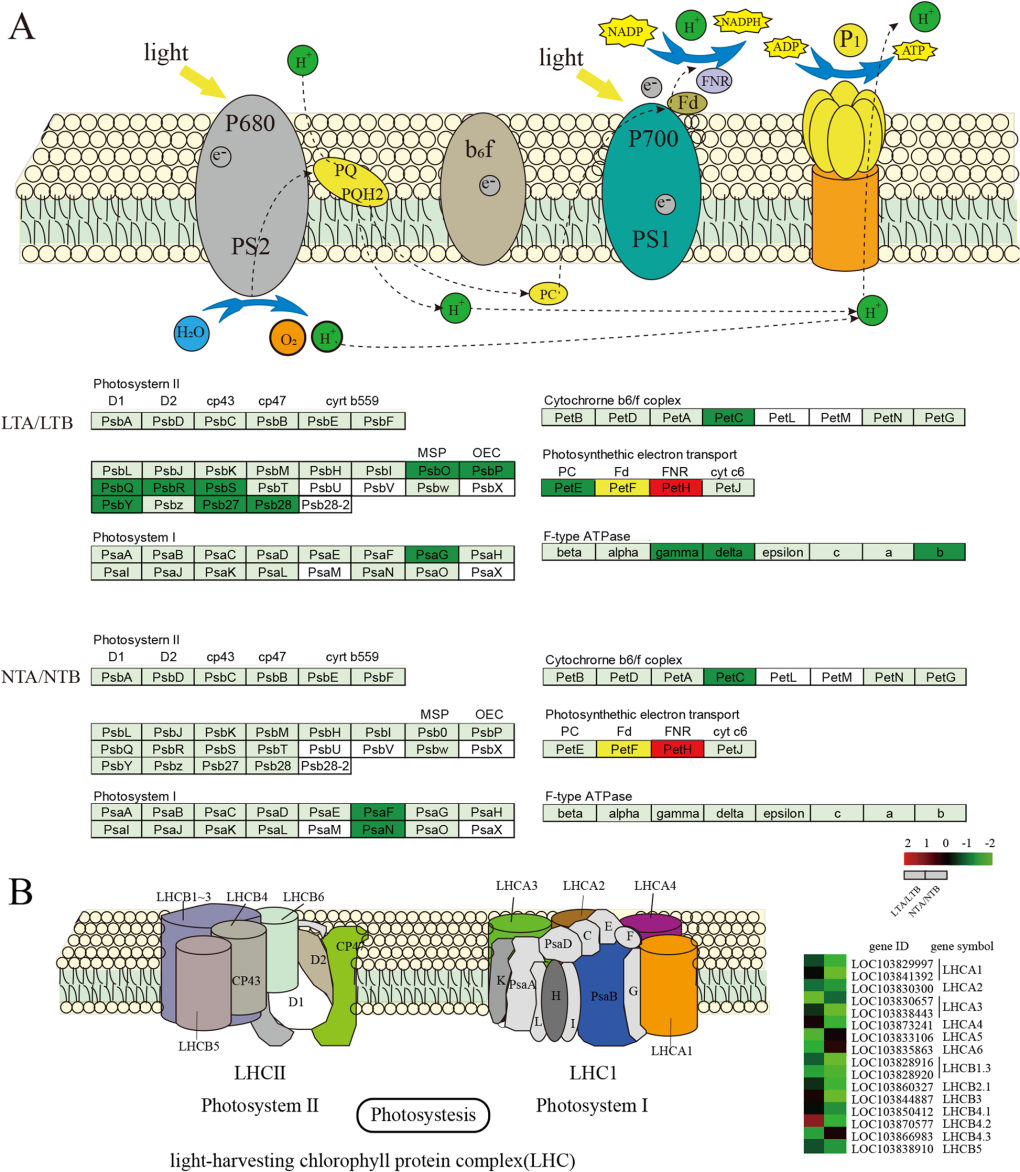
Heatmap of DEGs related to the photosynthesis (**A**) and photosynthesis-antenna proteins (**B**). LTA, low temperature after color change; LTB, low temperature before color change; NTA, normal temperature after color change; NTB, normal temperature before color change; LTA/LTB, with LTB as the control, DEGs were screened in LTA; NTA/NTB, with NTB as the control, DEGs were screened in NTA; *PsbO*, *photosystem II oxygen-evolving enhancer protein 1*; *PsbQ, photosystem II oxygen-evolving enhancer protein 3*; *PsbR*, *photosystem II protein PsbR*; *PsbS*, *photosystem II 22 kDa protein*; *PsbY*, *photosystem II core complex proteins psbY*; *Psb27*, *photosystem II protein Psb27*; *Psb28*, *photosystem II reaction center protein Psb28*; *PetC*, *photosynthetic electron transfer C*; *PsaO*, *photosystem I subunit O*; PetH, *ferredoxin-NADP oxidoreductase; PetE*, *plastocyanin*; *PsaF*, *photosystem I subunit F*; *PsaN*, *photosystem I subunit N*; *LHC*, *light-harvesting complex*

The genes involved in photosynthesis were mainly downregulated under LT, especially for the genes participating in PSII, PSI, and LHC (Fig. 10; Additional file 6: Table S6). In PSII, the DEGs included *photosystem II oxygen-evolving enhancer protein 1* (*PsbO*), *photosystem II oxygen-evolving enhancer protein 3* (*PsbQ*), *photosystem II protein PSBR* (*PsbR*)*, photosystem II 22 kDa protein* (*PsbS*), *photosystem II core complex proteins psbY* (*PsbY*), *photosystem II protein Psb27* (*Psb27*), and *photosystem II reaction center protein Psb28* (*Psb28*), and all these genes were decreased under LT. The expressions of *photosystem I subunit O* (*PsaO*), *photosynthetic electron transfer C* (*PetC*), *plastocyanin* (*PetE*), and f-type ATPase of subunits were downregulated under LT. The expression levels of *ferredoxin-NADP oxidoreductase* (*PetH*), *Photosystem I reaction center subunit III* (*PsaF*), *photosystem I subunit N* (*PsaN*), and *PetC* were downregulated under NT. Chl a/b binding proteins are important components of photochlorophyll-collecting protein complexes I and II, and most of genes encoding these proteins were downregulated under both LT and NT conditions. Specifically, five Chl a/b binding protein-encoding genes were decreased under LT, and 17 were decreased under NT (Fig. 10).

### DEGs of the circadian rhythm pathway

**Fig. 11:**
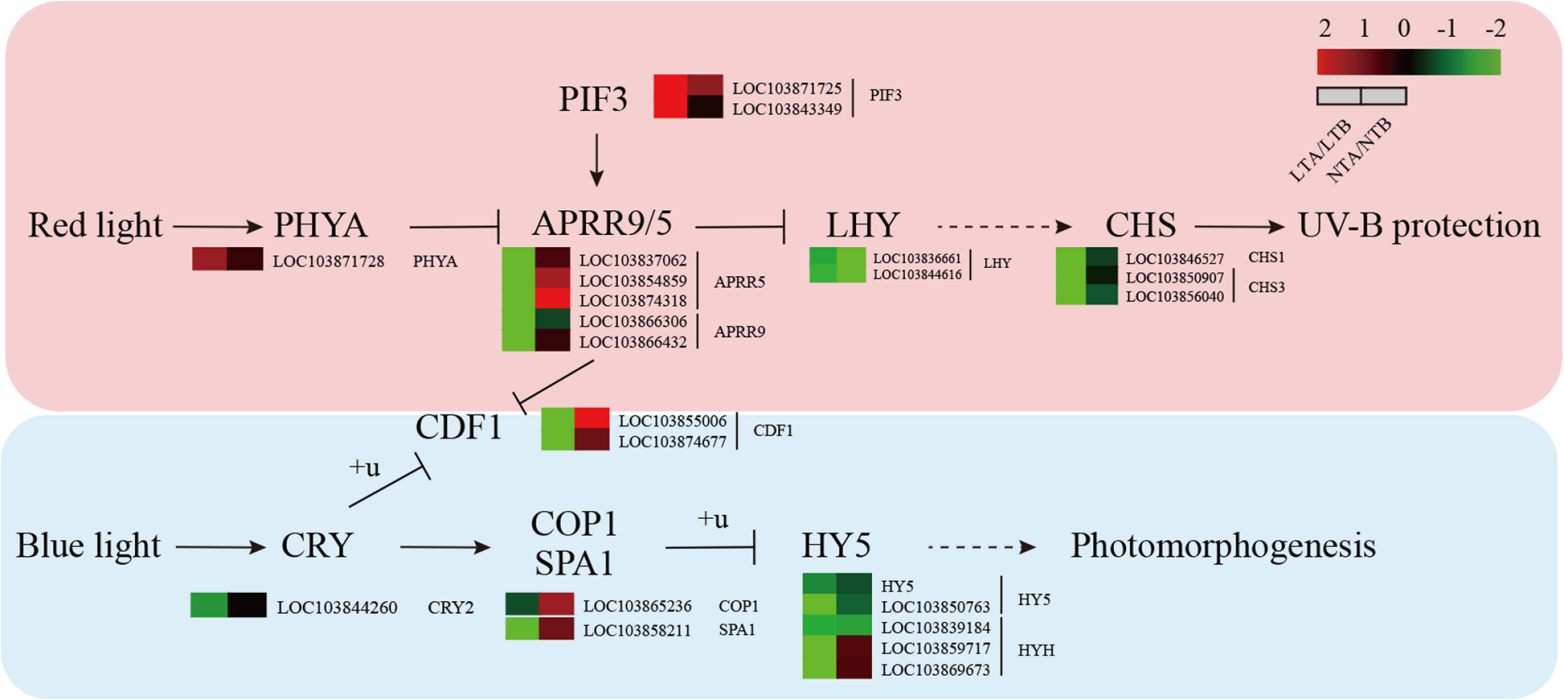
Heatmap of DEGs related to the circadian rhythm pathways. LTA, low temperature after color change; LTB, low temperature before color change; NTA, normal temperature after color change; NTB, normal temperature before color change; LTA/LTB, with LTB as the control, DEGs were screened in LTA; NTA/NTB, with NTB as the control, DEGs were screened in NTA; *PHYA*, *phytochrome A*; *LHY*, *LATE ELONGATED HYPOCOTYL*; *APRR*, *two-component response regulator*; *CHS*, *chalcone synthase*; *CRY*, *Cryptochromes*; *COP1*, *constitutive photomorphogenic 1*; *SPA1, SUPPRESSOR OF PHYA-105 1*; *HY5*, *LONG HYPOCOTYL5*; *CDF1*, *cyclic dof factor 1*

Genes involved in the circadian clock pathways can be divided into two pathways: the genes required for absorbing red and the genes required for absorbing blue light. A total of 34 DEGs were identified and analyzed (Figs. 11; Additional file 7: S7). Ten genes were upregulated and 21 were downregulated under LT, while eight were significantly upregulated and four were significantly downregulated under NT. Among them, two-component response regulator (*APRR*), chalcone synthase (*CHS*), *LATE ELONGATED HYPOCOTYL* (*LHY*), and cyclic dof factor 1 (*CDF1*) in the red-light pathway were downregulated under LT, but there were not differentially expressed under NT, except *LHY*. The expression of *LHY*, which plays an intermediate role in the red-light pathway, did not significantly differ between LT and NT. Cryptochrome circadian clock (*CRY*), *constitutive photomorphogenic 1* (*COP1*), protein SUPPRESSOR OF PHYA-105 1 (*SPA1*), and *LONG HYPOCOTYL5* (*HY5*) that participate in the blue light pathway were downregulated under LT, but their expressions were not affected under NT.

### The expression patterns of DEGs are verified by RT-qPCR

**Fig. 12:**
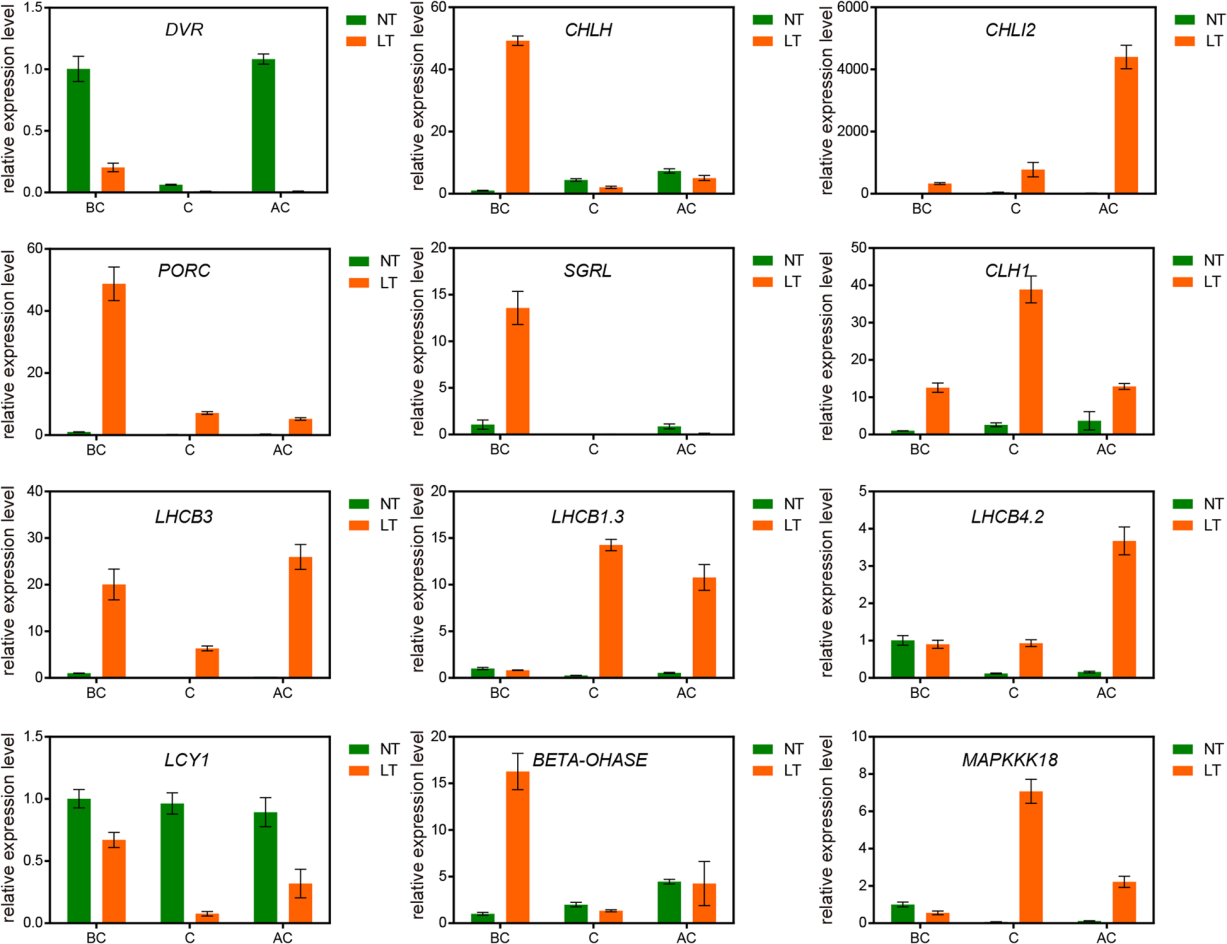
Verification of DEGs by using RT-qPCR assay. 12 DEGs were randomly selected and their transcript levels under both NT and LT conditions were analyzed by using qRT-PCR analysis. NT, normal temperature; LT, low temperature; BC, before color change; C, color change; AC, after color change. The line graphs represent the RT-qPCR data. Data are presented as the mean ± SD of three biological replicates

**Fig. 13:**
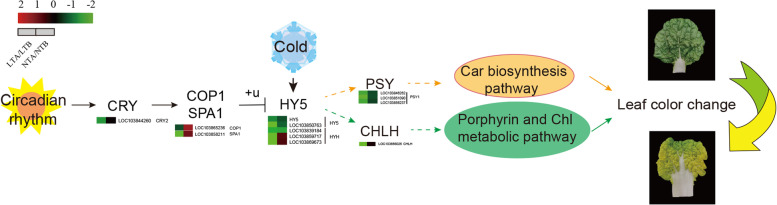
A proposed working model. In the circadian pathway in the response to blue light, *CRY* promotes the transcription of downstream genes *COP1* and *SPA1* after perceiving the blue light signal, which leads to the ubiquitination-mediated degradation of downstream gene *HY5*. Meanwhile, the transcript level of *HY5* is increased. Under low temperature condition, the expression of *HY5* is downregulated in wucai. *HY5* modulates the expression of two genes *CHLH* and *PSY*, which play a key role in the biosynthetic pathway of Chl and Car. The downregulation of *CHLH* and *PSY* leads to a reduced ratio of Chl/Car, which finally promotes leaf yellowing in wucai. The solid lines represent verified relationships, and the dashed lines indicate that the relationships need to be further investigated

To verify the RNA-Sequence (RNA-Seq) results, 12 DEGs were verified using quantitative reverse transcription PCR (RT-qPCR). These DEGs were involved in porphyrin and Chl metabolism (i.e. *CHLH, CHLI2, CLH1, DVR, PORC, SGRL*), Car biosynthesis (i.e. *BETA-OHASE, LCY1*), photosynthesis (i.e. *LHCB1.3, LHCB3, LHCB4.2*), and circadian circadian clock (i.e. *HY5*). Both the RNA-Seq and RT-qPCR assays showed the similar expression patterns of these DEGs, suggesting that the transcriptome data analyzed in this study were reliable (Figs. 12, 13; Additional file 3: Table S3).

## Discussion

Leaf pigmentation provides important information regarding the physiological state of plants. Chl plays a key role in photosynthetic reactions, while Car prevents damage to the photosynthetic system [18–20]. The content and composition of pigments vary greatly, which results in different leaf colors. Some of the low temperature-sensitive mutants exhibit a unique type of leaf discoloration. The mutant showed normal or nearly normal leaf color under NT, but different leaf color under LT. The formation of different leaf color is closely associated with photosynthetic pigments and photosynthesis. However, the molecular mechanism underlying the low temperatures-induced leaf color change remains unclear.

W7–2 is a low temperature-sensitive mutant [2]. The leaves of the W7–2 at the adult stage are green under normal temperatures but are yellow under low temperatures (Fig. 1). In order to identify the genes associated with leaf color change under LT, we used RNA-Seq to identify DEGs. This study focused on the study of DEGs in LTA/LTB and NTA/NTB groups to uncover the coloration mechanism. Through GO and KEGG analyses, we found that the enriched DEGs were associated with porphyrin and Chl metabolism, Car metabolism, photosynthesis, and circadian rhythm.

### The yellow leaf phenotype is closely associated with chlorophyll and carotenoids metabolism

Previous studies reported that plant leaf color is influenced by genetic factors and the external environment. These factors change the ratio of pigments (i.e. Chl and Car) that contribute to different leaf colors [21, 22]. Chl is mainly composed of blue-green Chl *a* and yellow-green Chl *b*. The contents of Chl and Car in W7–2 decreased significantly at NT and LT, and were lower at LT (Fig. 3). The ratio of Chl/Car in green leaves under NT was higher than that in yellow leaves under LT, which indicated that the b* value was higher under LT (Fig. 2B). These results verified that W7–2 is a low-temperature-sensitive cultivar, and its leaf color is sensitive to environmental temperatures. It was shown that the pigment content of plants would gradually decrease with senescence [8, 23]. Besides senescence, LT response is another factor that promotes the change of leaves from green to yellow.

Chl is an essential molecule to absorb solar energy in the photosynthetic antenna system [24]. It is also a necessary molecule in charge of the separation and electron transport of RCs. Chl exists in the thylakoid membrane as a pigment protein complex. The thylakoid components of the photosynthetic apparatus is crucial in regulating the temperature stress response in crop improvement [25]. Chl metabolism is a highly coordinated process catalyzed by a variety of enzymes. CHLH subunit is the largest subunit of magnesium chelatase, and the chelation of magnesium also takes place in H subunit [26, 27]. It catalyzes the transformation of protoporphyrin IX to Mg-protoporphyrin IX, which is a key step in Chl biosynthesis [11, 28]. Comparison of the transcriptome data of W7–2 under LT and NT conditions revealed that the expression of *CHLH* and *CHLI* under LT was significantly lower than that under NT (Fig. 8), which was consistent with the contents of Chl under LT and NT (Fig. 3). Analysis of seven intermediates involved in Chl biosynthesis indicated that the substrate level of Mg-chelatase was significantly reduced under LT (Fig. 4). These results suggested that the downregulation of *CHLH* at LT may reduce the activity of Mg-chelatase, thereby reducing the production of Chl *a* and *b*. The *CHLH* gene mutation leads to Chl deficiency, which thereby leads to green or yellow phenotypes in *Arabidopsis*, strawberry, and *Brassica napus* [29, 30]. The expression of *RCCR*, *Pao*, *MCS*, and *SGR1* genes, which encode the enzymes involved in the Chl degradation pathway, was not affected under LT and NT, indicating that the yellowing of leaves may due to the decrease in the biosynthesis of Chl.

Car coordinates with Chl in chloroplasts and is an important component in photosynthetic organs and photoprotective systems [31]. Car accumulates in plastids and forms different colors in plants [32–35], such as orange, yellow or red [36]. The Chl and Car contents and the total Chl/Car ratio of LT leaves were lower than NT at the same period (Fig. 3D, F). PSY and PDS are key enzymes in Car biosynthesis [37, 38]. The mutation of *PSY* and *PDS* can cause the decrease of carotenoid content [32, 39, 40]. In our study, the expression levels of *PSY* and *PDS* were significantly decreased under LT, which can explain the yellow leaves under LT.

### The lower photosynthetic efficiency of yellow leaf was regulated by the genes expression related to photosynthesis system

The color formation of yellow leaves is related to the development of chloroplasts, which includes the chloroplast membrane, thylakoid, and matrix, and is the main organelle of photosynthesis. Photosynthesis is one of the processes that are affected by low temperature [41]. It was postulated that photosynthesis and the development of thylakoids may be related to the changes in gene expression. LT can induce the expression of *PsbO* and *PsbP* in sugarcane [41]. PsbO is necessary for the binding of PSII and plays an important role in maintaining optimal evolutionary oxygen activity. Its mutation leads to the incomplete assembly of the PSII complex [19]. PsbP subunit is required for stabilizing the OEC structure [42]. Different from sugarcane, the expression of *PsbO* and *PsbP* in wucai was reduced, which may be related to the fact that wucai is a cold-tolerant vegetable. *Psb27* is very important for energy metabolism and the effective recovery of the PSII complex [41, 43, 44]. In this study, the *Psb27* expression level was decreased and the ETo/RC in PSII was also decreased under LT. The ETo/RC in Arabidopsis was significantly reduced after cold treatment, and the cold- responsive candidate gene *Psb27* may be one of the genes involved in the PSII in response to cold [44].

These parameters also reflect the changes in the PSII donor and recipient sides to some extent. When the donor side of PSII is injured, the Chl fluorescence intensity increases after a very short period of time, and the polyphase fluorescence O-J-I-P will become O-K-J-I-P at the K point (before the J point) [45–48]. In our study, the K point did not appear under NT or LT, indicating that the two temperature treatments did not adversely affect wucai growth. φPo and Fv/Fm reflect the maximum photochemical efficiency of leaves [49]. In our study, the maximum photochemical efficiency of green leaves at NT was higher than yellow leaves at LT. Sm reflects the size of the electron acceptor pool on the PSII acceptor side of the leaves [49]. Sm was decreased at LT, resulting in a decrease in quantum yield for electron transfer. Part of the energy absorbed by the antenna pigment (Chl) is dissipated in the form of heat and fluorescence. Such dissipation is a kind of protection for plants. The other part of the energy was captured and converted into reduction energy, reducing QA to QA-. QA- can be re-oxidized to generate electrons for carbon dioxide fixation [50]. In our research, the amount of energy absorption, dissipation and the quantum yield of electron transfer per leaf area decreased under LT, and increased under NT (Fig. 5). The lower ET_O_/CS_O_ indicated that the ability for carbon dioxide fixation was weaker at LT.

### HY5 involved in the regulation of yellow leaf phenotype responding to cold

The circadian rhythm pathway can be divided into the red and blue light pathways. After *PHYA* receiving red light (RL) at LT, the transcription level increases, which leads to an enhanced inhibitory effect on the downstream gene *APRR5/9*, and regulates UV-B protection. *CRY* promotes the expression of *COP1* and *SPA1* after receiving blue light signals. Blue light signal can induce its own cascade pathway, which may inhibit the expression of *COP1* by affecting the activity of *CRY1/2*, resulting in an increase in the transcript level of downstream genes [51]. *COP1* and *SPA1* can induce the ubiquitination-mediated degradation of *HY5* and *HYH*, thereby regulating photomorphogenesis. What is interesting is our study showed that that even after COP1 and SPA1 were downregulated, the expression of downstream transcript factor HY5 and HYH, which should be upregulated, were also downregulated under LT, indicating that in addition to the circadian rhythm, low temperature can also affect their expression. *HY5* is involved in LT response and regulates the expression of several cold-induced genes [52]. Here we speculated that LT is also an important factor that affects the expression of *HY5*. LT significantly and rapidly upregulates the expression of *SlHY5* in tomato seedlings and *LaHY5* in *Monochamus chinensis* seedlings [53], but the underlying molecular mechanism of how LT regulates the Chl and Car biosynthesis pathways through *HY5* has not been elucidated or investigated. In our study, we found that LT altered the color of leaves by reducing the expression level of *HY5*, which subsequently regulates the downstream key genes, *CHLH* and *PSY* that participate in the Chl and Car biosynthesis pathways in wucai.

## Materials and methods

### Plant materials and growth conditions

W7–2, is a new wucai cultivar line that possesses green inner leaves under normal temperatures and turned yellow during the growing period under low temperature [2, 16]. The plant material was stabilized by inheritance through multiple generations. The cultivar is widely planted throughout the Yangtze-Huai River Basin in China.

This experiment was conducted in the breeding basement of Anhui Agricultural University, Hefei, China. W7–2 seedlings were planted in a greenhouse at 26 ± 2 °C (day) and 20 ± 1.5 °C (night) with 70–75% relative humidity. Seedlings with 4–5 leaves were transplanted in pots (0 days after planting (DAP)) then transferred to a growth chamber, while seedlings with 7–8 leaves were transferred to an ultra-low temperature growth chamber. After being transferred to the ultra-low temperature incubator, a pre-hardening step is performed first, and the temperature was set to 8 °C from 0:00 to 7:30, 11 °C from 7:30 to 10:30, 16 °C from 10:30 to 14:30, 11 °C from 14:30 to 19:30, and 8 °C from 19:30 to 24:00 with a light intensity of 300 μmol m^− 2^ s^− 1^. After 2 days’ pre-hardening, the temperature of the ultra-low temperature growth chamber dropped six degrees. The chamber parameters were in accordance with the local weather. The temperature of the growth chamber was set to 26 ± 2 °C (day) and 18 ± 2 °C (night) with a light intensity of 300 μmol m^− 2^ s^− 1^. After moving into the chambers, the third fully expanded young leaves from the center of the plants were sampled every 3 days. Samples were immediately frozen in liquid nitrogen and maintained at − 80 °C for further physiological and biochemical analyses.

### Measurement of color values

Leaf color values were measured using a CR-400-C chroma meter (Konica Minolta Sensing Americas, Inc., Ramsey, MN, USA) using the upper surface of the leaves every 2 DAP. In both treatments, five plants were measured, and measurements were repeated three times in the same leaf position. The data were analyzed using the CIELAB color coordinate system [2]. There were three main leaf color values: Value L*, Value a*, and Value b*. “L*” represents the brightness of the object, where 0–100 represents black to white. “a*” represents the red and green of the object, where a positive value indicates red and a negative value indicates green. “b*” represents the yellow and blue of the object, where a positive value indicates yellow and a negative value indicates blue.

### Measurement of chlorophyll and carotenoids content

The contents of photosynthetic pigments (Chl a, Chl b, and Car) were estimated using the previously described methods of Arnon [54] with slight modifications. Fresh leaves were ground to powder, then 0.2 g was mixed in a 50 mL solvent mixture (acetone/V: ethanol/V: water/V = 4.5:4.5:1) for 24 h at ~ 4 °C in the dark. The absorbance at wavelengths of 470, 646, and 663 nm was measured by a TU1950 UV-vis spectrophotometer (PERSEE, Beijing, China). Chl a (mg/g), Chl b (mg/g), and Car (mg/g) contents were calculated as follows:

$${\mathrm{C}}_{\mathrm{C}\mathrm{hl}\ \mathrm{a}}\ \left(\mathrm{mg}/\mathrm{L}\right)=12.21\times {\mathrm{A}}_{663}-2.81\times {\mathrm{A}}_{646};$$

$${\mathrm{C}}_{\mathrm{C}\mathrm{hl}\ \mathrm{b}}\ \left(\mathrm{mg}/\mathrm{L}\right)=20.13\times {\mathrm{A}}_{646}-5.03\times {\mathrm{A}}_{663};$$

$${\mathrm{C}}_{\mathrm{C}\mathrm{ar}}\ \left(\mathrm{mg}/\mathrm{L}\right)=4.37\times {\mathrm{A}}_{470}+2.11\times {\mathrm{A}}_{663}-9.10\times {\mathrm{A}}_{646};$$

$$\mathrm{Chl}\ \mathrm{a}\ \left(\mathrm{mg}/\mathrm{g}\right)={\mathrm{C}}_{\mathrm{C}\mathrm{hl}\ \mathrm{a}}\ \left(\mathrm{mg}/\mathrm{L}\right)\times \mathrm{V}\ \left(\mathrm{L}\right)/{\mathrm{M}}_{\mathrm{fresh}}\ \left(\mathrm{g}\right);$$

$$\mathrm{Chl}\ \mathrm{b}\ \left(\mathrm{mg}/\mathrm{g}\right)={\mathrm{C}}_{\mathrm{C}\mathrm{hl}\ \mathrm{b}}\ \left(\mathrm{mg}/\mathrm{L}\right)\times \mathrm{V}\ \left(\mathrm{L}\right)/{\mathrm{M}}_{\mathrm{fresh}}\ \left(\mathrm{g}\right);$$

$$\mathrm{Car}\ \left(\mathrm{mg}/\mathrm{g}\right)={\mathrm{C}}_{\mathrm{car}}\ \left(\mathrm{mg}/\mathrm{L}\right)\times \mathrm{V}\ \left(\mathrm{L}\right)/{\mathrm{M}}_{\mathrm{fresh}}\ \left(\mathrm{g}\right);$$

$$\mathrm{Chl}\ \mathrm{a}/\mathrm{b}\ \mathrm{ratio}=\mathrm{Chl}\ \mathrm{a}/\mathrm{Chl}\ \mathrm{b}.$$

### Measurement of Chl metabolism

The Glu content was measured using a Solarbio reagent kit (Cat #BC1580; Beijing Solarbio Science & Technology Co., Ltd., Beijing, China). To determine the ALA content, we used Morton’s method with slight modifications. A total of 0.5 g fresh sample was added to 2 mL 0.5 mol/L extraction solution (pH = 4.6) after grinding in an ice bath, boiling in a water bath for 15 min, and centrifuging at 10,000 g for 15 min. Then, 1 mL supernatant was mixed with 50 μL acetylacetone and bathed in boiling water. OD_553_ was determined after dark treatment for 15 min. To determine the PBG concentration, we used Bogorad’s method [55] with slight modifications.

The protoporphyrin IX, Mg-protoporphyrin IX, and Pchlide contents were measured using Hodgins’s method [56] with slight modifications. A total of 0.5 g fresh sample was added to 5 mL 80% alkaline acetone. The mixture was ground, and the volume was fixed to 25 mL. Then, the mixture was centrifuged at 12,000 g for 10 min at 4 °C. The supernatant was used to measure the absorbance at 575, 590, and 628 nm.

The Urogen III and Coprogen III contents were measured using Bogorad’s method [55] with slight modifications. A total of 0.2 g fresh sample was ground with liquid nitrogen, transferred to a centrifuge tube with 3 mL 0.067 M (pH = 6.8) phosphate buffer, and centrifuged at 12,000 g for 10 min. Then, 1.5 mL supernatant was added to 75 μL 1% Na_2_S_2_O_3_ after vigorous shaking and exposed to strong light for 20 min. The pH was adjusted to 3.5 with acetic acid. OD_405.5_ was measured after extraction with ethyl ether, and OD_399.5_ was measured after extraction with HCl.

### Chl fluorescence parameter analysis

In vivo Chl fluorescence emissions were measured in 30 min dark-adapted leaves with Handy PEA *(Hansatech Instruments Ltd.,* Norfolk, *UK)*. The data were sampled at 10 μs intervals for the first 300 μs, providing an excellent time resolution of Fo and the initial rise kinetics. Chl a fluorescence emission was induced between 10 μs and 1 s by digitized light pulses produced by the instrument. Chl a fluorescence transient was analyzed using the JIP-test formula. The fluorescence intensity at 20 μs was considered Fo. At this time, the fluorescence intensity was considered the minimum fluorescence after dark adaptation when all RCs were completely open and the maximum fluorescence intensity was presumed to be equal to Fm (Fp). The intensity was sufficient to ensure the closure of all RCs of PSII, namely, the maximum fluorescence after dark adaptation. Moreover, the fluorescence intensity at 300 μs (F300 μs), 2 ms (J-step, FJ), and 30 ms (I-step, FI) was measured.

The following parameters refer to time 0 (Fo): the specific energy fluxes of absorption per RC (ABS/RC), trapping (TRo/RC), electron transport (ETo/RC), and dissipation at the level of antenna Chl (DIo/RC) (Fig. 6C). The specific energy fluxes of absorption per excited cross section (ABS/CS), trapping (TRo/CS), electron transport (ETo/CS), and dissipated energy flux per excited cross section (DIo/CS) were also measured. Other parameters measured in this study are provided (Table S2).

### Transcriptome analysis and gene annotation

The total RNA of 18 samples from six treatments were extracted using a mirVana™ miRNA Isolation Kit (Ambion, Austin, TX, USA) following the manufacturer’s instructions. RNA integrity was evaluated using an Agilent 2100 Bioanalyzer (Agilent Technologies, Santa Clara, CA, USA). Samples with an RNA integrity number (RIN) ≥ 7 were subjected to subsequent analysis. Libraries were constructed using a TruSeq Stranded mRNA LT Sample Prep Kit (Illumina, San Diego, CA, USA) following the manufacturer’s instructions. Then, the libraries were sequenced on an Illumina sequencing platform (HiSeq TM 2500 or Illumina HiSeq X Ten), and 125 or 150 bp paired-end reads were generated. After quality inspection, the Illumina sequencer was used for sequencing. In order to obtain high-quality clean reads that can be used for subsequent analysis, Trimmomatic [57] was used for quality control; joints, low-quality bases, and n-bases were removed. The Clean, filtered reads were aligned to the *B. rapa* reference genome by HISAT2 [58]. The gene Fragments Per Kilobase of transcript per Million mapped reads (FPKM) values were quantified by Cuff software [59, 60]. For the gene expression differences calculations, HtSeq count was used to obtain the number of reads of genes falling into each sample [61]. The estimated size factors function using the “DESeq” R package was used to standardize the data [62]. The “nbinom Test” function was used to calculate the foldchange and *p*-values. False discovery rate (FDR) was used to account for multiple comparisons. DEGs with a p-value < 0.05 and |log2(foldchange)| > 1 were selected. GO and KEGG enrichment analyses of the DEGs were conducted to determine the biological functions or pathways mainly affected by the DEGs. Then, the DEGs were clustered by unsupervised hierarchical clustering, and their expression patterns in different samples were displayed in the form of heat maps.

### Validation of gene expression

To verify the transcript levels of related genes after color conversion in W7–2 leaves using the transcriptome data, genes related to Chl biosynthesis (i.e. *CHLH, CHLI2, CLH1, DVR, PORC, SGRL*), Car metabolism (i.e. *BETA-OHASE, LCY1*), photosynthesis (i.e. *LHCB1.3, LHCB3, LHCB4.2*), and circadian clock (i.e. *HY5*) were further analyzed by RT-qPCR. The total RNA was isolated from LTB, NTB, LTA, and NTA using a total RNA kit (Takara Biomedical Technology Co., Beijing, China). Primer software v6.0 (Premier Biosoft International, Palo Alto, CA, USA) was used to design specific gene primers (Table S3). The gene encoding actin was used as the control. RT-qPCR was performed using SYBR GREEN Master Mix (Vazyme Biotechnology Co., Ltd., Nanjing, China). Relative gene expression levels were calculated using the 2^-ΔΔCT^ method [63].

### Statistical analysis

All data are presented as the mean ± SD using at least three biological replicates. SPSS v22.0 (SPSS Institute Inc., Chicago, IL, USA) was used for the statistical analyses. Tukey’s post-hoc test was used for mean comparisons using *p* < 0.05. Graphics were drawn using GraphPad Prism software (*GraphPad* Software Inc., *USA*), Origin 2018 64Bit, and Adobe Illustrator CC 2019.

## Supplementary Information

**Additional file 1: Table S1.** Summary of transcriptome sequencing data obtained using Illumina technology.

**Additional file 2: Table S2.** Chlorophyll fluorescence parameters in wucai leaves under LT and NT.

**Additional file 3: Table S3.** The primer sequences used in RT-qPCR.

**Additional file 4: Table S4.** DEGs of porphyrin and Chl metabolism pathways.

**Additional file 5: Table S5.** DEGs of carotenoid biosynthesis and abscisic acid biosynthesis pathways.

**Additional file 6: Table S6.** DEGs of photosynthesis and the photosynthesis-antenna pathway.

**Additional file 7: Table S7.** DEGs of the circadian rhythm pathway.

## Data Availability

The RNA-Seq datasets are available in the Sequence Read Archive of National Center for Biotechnology Information (https://dataview.ncbi.nlm.nih.gov/object/PRJNA687807?reviewer=u796b8k88hqfkpp6b5pqv4tkl1; accession number: PRJNA687807).

## Abbreviations

LT: Low temperature
NT: Normal temperature
BC: Before color change
C: Color change
AC: After color change
LTA: Low temperature after color change
LTC: Low temperature during color change
LTB: Low temperature before color change
NTA: Normal temperature after color change
NTB: Normal temperature before color change
LTA/LTB: With LTB as the control, DEGs were screened in LTA
NTA/NTB: With NTB as the control, DEGs were screened in NTA
DAP: Day after planting
Chl: Chlorophyll
Car: Carotenoids
ALA: 5-aminolevulinic acid
PBG: Porphobilinogen
Pchl: Protochlorophyllide
Proto IX: Protoporphyrin IX
Mg-proto IX: Mg-protoporphyrin IX
Urogen III: Uroporphyrinogen III
Coproen III: Coproporphyrin III
DEG: Differentially expressed gene
GO: Gene Ontology
KEGG: Kyoto Encyclopedia of Genes and Genomes
PSI: Photosystem I
PSII: Photosystem II
RPKM: The reads per kilo bases per million read
Fm: Maximum fluorescence
Fv: Variable fluorescence
φPo: Maximum quantum yield for primary photochemistry
Sm: Normalised total complementary area above the O-J-I-P transie
φEo: Quantum yield for electron transport
RC: Reaction centre
RC/CS: The concentration of active PSII reaction centres per excited cross section
ABS/RC: Absorption of flux per RC
DIo/RC: Dissipated energy flux per RC
TRo/RC: Trapped energy flux per RC
ETo/RC: Electron transport flux per RC
REo/RC: Electron transfer per RC to the end of PSI
ABS/DIo: Absorption/ dissipated energy
ABS/CSo: Absorption of flux per CS
DIo/CSo: Dissipated energy flux per CS
TRo/CSo: Trapped energy flux per CS
ETo/CSo: Electron transport flux per CS
REo/CSo: Electron transfer per CS to the end of PSI
*GluRS*: *Glutamyl-tRNA synthetase*
*HEMA*: *Glutamyl-tRNA reductase*
*HEML*: *Glutamate-1-semialdehyde 2,1-aminomutase*
*HEMB*: *Porphobilinogen synthase*
*HEMC*: *Hydroxymethylbilane synthase*
*HEMD*: *Uroporphyrinogen-III synthase*
*HEME*: *Uroporphyrinogen decarboxylase*
*HEMF*: *Coproporphyrinogen III oxidase*
*CHLH*: *Magnesium chelatase subunit H*
*CHLM*: *Magnesium-protoporphyrin IX methyltransferase*
*DVR*: *3,8-Divinyl protochlorophyllide a 8-vinyl reductase*
*CRD*: *Chloroplast Relocation Defective*
*POR*: *NADPH-protochlorophyllide oxidoreductase*
*CAO*: *Chlorophyllide a oxygenase*
*CHLG*: *Chlorophyll/bacteriochlorophyll a synthase*
*CLH*: *Chlorophyllase*
*CHLP*: *Geranylgeranyl reductase*
*HCAR*: *7-hydroxymethyl chlorophyll a reductase*
*NOL*: *Non-yellow coloring-like*
*NYC1*: *Non-yellow coloring*
*SGRL*: *Stay-Green Rice like*
*PaO*: *Pheophorbide a oxygenase*
*RCCR*: *Red chlorophyll catabolite reductase*
*PSY*: *Phytoene synthase*
*PDS*: *Phytoene desaturase*
*Z-ISO*: *Zeta-carotene isomerase*
*ZDS*: *ζ- carotene desaturase*
*CRTISO*: *Carotene Cis-Trans Isomerase*
*LCY*: *Lycopene beta cyclas*e
*LUT5*: *Enhanced five-input lookup table*
*BETA-OHASE*: *Beta-carotene 3-hydroxylase 1*
*PsbO*: *Photosystem II oxygen-evolving enhancer protein 1*
*PsbQ*: *Photosystem II oxygen-evolving enhancer protein 3*
*PsbR*: *Photosystem II protein PsbR*
*PsbS*: *Photosystem II 22 kDa protein*
*PsbY*: *Photosystem II core complex proteins psbY*
*Psb27*: *Photosystem II protein Psb27*
*Psb28*: *Photosystem II reaction center protein Psb28*
*PetC*: *Photosynthetic electron transfer C*
*PsaO*: *Photosystem I subunit O*
PetH: *Ferredoxin-NADP oxidoreductase*
*PetE*: *Plastocyanin*
*PsaF*: *Photosystem I subunit F*
*PsaN*: *Photosystem I subunit N*
*LHC*: *Light-harvesting complex*
*PHYA*: *Phytochrome A*
*LHY*: *LATE ELONGATED HYPOCOTYL*
*APRR*: *Two-component response regulator*
*CHS*: *Chalcone synthase*
*CRY*: *Cryptochromes*
*COP1*: *Constitutive photomorphogenic 1*
*SPA1*: *SUPPRESSOR OF PHYA-105 1*
*HY5*: *LONG HYPOCOTYL5*
*CDF1*: *Cyclic dof factor 1*
