# Supplementary material for: Comparative transcriptome analysis reveals that chlorophyll metabolism contributes to leaf color changes in wucai (Brassica campestris L.) in response to cold
Source: BMC Plant Biol. 2021 Sep 28;21:438. doi: 10.1186/s12870-021-03218-9 (PMC8477495; doi:10.1186/s12870-021-03218-9)
Supplement: Supplementary file 1 — Additional file 1: Table S1. Summary of transcriptome sequencing data obtained using Illumina technology. [file 12870_2021_3218_MOESM1_ESM.docx]

**Comparative Transcriptome Analysis Reveals that Chlorophyll Metabolism Contributes to Leaf Color Changes in Wucai (*Brassica campestris* L.) in Response to Cold**

Lingyun Yuan ^1,2,3†^, Liting Zhang ^1,2†^, Ying Wu ^1,2^, Yushan Zheng ^1,2^, Libing Nie ^1,2^, Shengnan Zhang ^1,2^, Tian Lan ^1,2^, Yang Zhao ^1,2^, Shidong Zhu ^1,2,3^, Jinfeng Hou ^1,2,3^, Guohu Chen ^1,2,3^, Xiaoyan Tang ^1,2,3^ and Chenggang Wang ^1,2,3*^

^†^These authors contributed equally to this work.

^*^Corresponding author: Chenggang Wang

Tel./Fax. +86 0551-65786212

E-mail: cgwang@ahau.edu.cn

^1^College of Horticulture, Vegetable Genetics and Breeding Laboratory, Anhui Agricultural University, 130 West Changjiang Road, 230036 Hefei, Anhui, China;

^2^Provincial Engineering Laboratory for Horticultural Crop Breeding of Anhui, 130 West of Changjiang Road, 230036 Hefei, Anhui, China;

^3^Wanjiang Vegetable Industrial Technology Institute, Maanshan, Anhui, 238200, China

Table S1

| Sample | LTB1 | LTB2 | LTB3 | LTC1 | LTC2 | LTC3 | LTA1 | LTA2 | LTA3 | NTB1 | NTB2 | NTB3 | NTC1 | NTC2 | NTC3 | NTA1 | NTA2 | NTA3 |
| --- | --- | --- | --- | --- | --- | --- | --- | --- | --- | --- | --- | --- | --- | --- | --- | --- | --- | --- |
| Raw reads (M) | 41.49 | 52.50 | 57.62 | 54.16 | 57.18 | 52.41 | 50.70 | 57.39 | 50.22 | 51.20 | 49.52 | 41.10 | 52.20 | 52.33 | 50.98 | 56.07 | 50.10 | 54.47 |
| Raw bases (G) | 6.22 | 7.88 | 8.64 | 8.12 | 8.58 | 7.86 | 7.61 | 8.61 | 7.53 | 7.68 | 7.43 | 6.17 | 7.83 | 7.85 | 7.65 | 8.41 | 7.52 | 8.17 |
| Clean reads (M) | 40.85 | 51.34 | 56.70 | 53.18 | 56.01 | 51.65 | 49.84 | 56.37 | 49.30 | 50.07 | 48.47 | 40.23 | 51.21 | 51.50 | 50.07 | 55.06 | 49.37 | 53.53 |
| Clean bases (G) | 5.95 | 7.05 | 8.24 | 7.33 | 7.74 | 7.47 | 6.86 | 7.78 | 6.93 | 6.85 | 6.77 | 5.61 | 7.27 | 7.14 | 7.07 | 7.73 | 7.15 | 7.59 |
| Valid bases (%) | 95.64 | 89.46 | 95.36 | 90.21 | 90.25 | 94.97 | 90.18 | 90.39 | 91.99 | 89.18 | 91.21 | 90.98 | 92.90 | 90.94 | 92.51 | 91.90 | 95.17 | 92.85 |
| Q30 (%) | 95.31 | 94.19 | 95.21 | 94.86 | 94.49 | 95.33 | 94.44 | 94.80 | 94.48 | 94.48 | 94.42 | 94.58 | 94.73 | 94.72 | 94.81 | 94.63 | 95.36 | 94.88 |
| GC (%) | 47.60 | 47.37 | 47.07 | 46.80 | 46.28 | 46.29 | 46.34 | 46.63 | 46.83 | 47.37 | 47.49 | 47.31 | 47.48 | 47.06 | 47.28 | 47.24 | 47.21 | 47.18 |

Summary of transcriptome sequencing data obtained using Illumina technology.
