# Supplementary material for: Comparative transcriptome analysis reveals that chlorophyll metabolism contributes to leaf color changes in wucai (Brassica campestris L.) in response to cold
Source: BMC Plant Biol. 2021 Sep 28;21:438. doi: 10.1186/s12870-021-03218-9 (PMC8477495; doi:10.1186/s12870-021-03218-9)
Supplement: Supplementary file 2 — Additional file 2: Table S2. Chlorophyll fluorescence parameters in wucai leaves under LT and NT. [file 12870_2021_3218_MOESM2_ESM.docx]

**Comparative Transcriptome Analysis Reveals that Chlorophyll Metabolism Contributes to Leaf Color Changes in Wucai (*Brassica campestris* L.) in Response to Cold**

Lingyun Yuan ^1,2,3†^, Liting Zhang ^1,2†^, Ying Wu ^1,2^, Yushan Zheng ^1,2^, Libing Nie ^1,2^, Shengnan Zhang ^1,2^, Tian Lan ^1,2^, Yang Zhao ^1,2^, Shidong Zhu ^1,2,3^, Jinfeng Hou ^1,2,3^, Guohu Chen ^1,2,3^, Xiaoyan Tang ^1,2,3^ and Chenggang Wang ^1,2,3*^

^†^These authors contributed equally to this work.

^*^Corresponding author: Chenggang Wang

Tel./Fax. +86 0551-65786212

E-mail: cgwang@ahau.edu.cn

^1^College of Horticulture, Vegetable Genetics and Breeding Laboratory, Anhui Agricultural University, 130 West Changjiang Road, 230036 Hefei, Anhui, China;

^2^Provincial Engineering Laboratory for Horticultural Crop Breeding of Anhui, 130 West of Changjiang Road, 230036 Hefei, Anhui, China;

^3^Wanjiang Vegetable Industrial Technology Institute, Maanshan, Anhui, 238200, China

Table S2

Chlorophyll fluorescence parameters in wucai leaves under LT and NT.

| parameters | NTB | LTB | NTA | LTA |
| --- | --- | --- | --- | --- |
| Fm | 1524 | 1425 | 1550 | 1439 |
| Fv/Fm | 0.847769029 | 0.818947368 | 0.827096774 | 0.820708826 |
| phi(Po) | 0.8543 | 0.8218 | 0.852 | 0.8416 |
| Sm | 40.6933 | 68.4528 | 53.4433 | 57.5216 |
| phi(Eo) | 0.626 | 0.6484 | 0.5613 | 0.6129 |
| ABS/RC | 0.7848 | 1.1988 | 1.1042 | 1.0835 |
| DIo/RC | 0.1143 | 0.2137 | 0.2251 | 0.1717 |
| TRo/RC | 0.6705 | 0.9852 | 0.8791 | 0.9119 |
| ETo/RC | 0.4913 | 0.7774 | 0.6198 | 0.6641 |
| REo/RC | 0.211 | 0.506 | 0.335 | 0.358 |
| ABS/CSm | 1524 | 1425 | 1550 | 1439 |
| DIo/CSm | 222 | 254 | 316 | 228 |
| TRo/CSm | 1302 | 1171 | 1234 | 1211 |
| ETo/CSm | 954 | 924 | 870 | 882 |
| REo/CSm | 410 | 602 | 470 | 475 |
