# Supplementary material for: Comparative transcriptome analysis reveals that chlorophyll metabolism contributes to leaf color changes in wucai (Brassica campestris L.) in response to cold
Source: BMC Plant Biol. 2021 Sep 28;21:438. doi: 10.1186/s12870-021-03218-9 (PMC8477495; doi:10.1186/s12870-021-03218-9)
Supplement: Supplementary file 3 — Additional file 3: Table S3. The primer sequences used in RT-qPCR. [file 12870_2021_3218_MOESM3_ESM.docx]

**Comparative Transcriptome Analysis Reveals that Chlorophyll Metabolism Contributes to Leaf Color Changes in Wucai (*Brassica campestris* L.) in Response to Cold**

Lingyun Yuan ^1,2,3†^, Liting Zhang ^1,2†^, Ying Wu ^1,2^, Yushan Zheng ^1,2^, Libing Nie ^1,2^, Shengnan Zhang ^1,2^, Tian Lan ^1,2^, Yang Zhao ^1,2^, Shidong Zhu ^1,2,3^, Jinfeng Hou ^1,2,3^, Guohu Chen ^1,2,3^, Xiaoyan Tang ^1,2,3^ and Chenggang Wang ^1,2,3*^

^†^These authors contributed equally to this work.

^*^Corresponding author: Chenggang Wang

Tel./Fax. +86 0551-65786212

E-mail: cgwang@ahau.edu.cn

^1^College of Horticulture, Vegetable Genetics and Breeding Laboratory, Anhui Agricultural University, 130 West Changjiang Road, 230036 Hefei, Anhui, China;

^2^Provincial Engineering Laboratory for Horticultural Crop Breeding of Anhui, 130 West of Changjiang Road, 230036 Hefei, Anhui, China;

^3^Wanjiang Vegetable Industrial Technology Institute, Maanshan, Anhui, 238200, China

Table S3.

The primer sequences used in RT-qPCR.

| Gene name | Primer name | Primer sequence (5’->3’) |
| --- | --- | --- |
| *BnaActin* | primer F | TGGGTTTGCTGGTGACGAT |
|  | primer R | TGCCTAGGACGACCAACAATACT |
| *BETA-OHASE* | primer F | CGGTACATTCGCTCTATCTGTTG |
|  | primer R | CGTTGACTATGGCGAACACA |
| *CHLH* | primer F | TTGGAAGGCAGATTGAAGAT |
|  | primer R | CTGTTGGCACGGTAGAAT |
| *CHLI2* | primer F | CGCAAGTAGGCACAGTTA |
|  | primer R | TCACCTCTCAGTCCATCAA |
| *CLH1* | primer F | GGAACTTACCCCGTCGTCT |
|  | primer R | ATCACTTTTCCAGCATCGTC |
| *DVR* | primer F | AACTCCTCTCACGACCTC |
|  | primer R | GAACCACGAATCTGCCTAT |
| *HY5* | primer F | TCTTTACCTGCAAGCTCCC |
|  | primer R | TCCAGAGATTTCCTTTCCGGT |
| *LCY1* | primer F | ACCGTAAACAGCTCAAATCCAAG |
|  | primer R | CGACCTCAGCAACGATACCAT |
| *LHCB1.3* | primer F | TTTGGCTATTTGGGCTACTC |
|  | primer R | ACATAGCCAATCTTCCGTTC |
| *LHCB3* | primer F | TGATGGTGTTGGTGAAGG |
|  | primer R | TCAAGATGGTCAAGGAGATT |
| *LHCB4.2* | primer F | ATCCAGGAGGCAAGTTCT |
|  | primer R | CGATGATGGTGGTGTGAA |
| *PORC* | primer F | CACGTACCGCTGTTTAGGCTT |
|  | primer R | TCTCCCACAGTTTCTTTGCCTT |
| *SGRL* | primer F | TGGTTGCTGAGTGGAAGA |
|  | primer R | TTAGGTGTGCTTGAATGGAA |
