# Supplementary material for: Comparative transcriptome analysis reveals that chlorophyll metabolism contributes to leaf color changes in wucai (Brassica campestris L.) in response to cold
Source: BMC Plant Biol. 2021 Sep 28;21:438. doi: 10.1186/s12870-021-03218-9 (PMC8477495; doi:10.1186/s12870-021-03218-9)
Supplement: Supplementary file 4 — Additional file 4: Table S4. DEGs of porphyrin and Chl metabolism pathways. [file 12870_2021_3218_MOESM4_ESM.docx]

**Comparative Transcriptome Analysis Reveals that Chlorophyll Metabolism Contributes to Leaf Color Changes in Wucai (*Brassica campestris* L.) in Response to Cold**

Lingyun Yuan ^1,2,3†^, Liting Zhang ^1,2†^, Ying Wu ^1,2^, Yushan Zheng ^1,2^, Libing Nie ^1,2^, Shengnan Zhang ^1,2^, Tian Lan ^1,2^, Yang Zhao ^1,2^, Shidong Zhu ^1,2,3^, Jinfeng Hou ^1,2,3^, Guohu Chen ^1,2,3^, Xiaoyan Tang ^1,2,3^ and Chenggang Wang ^1,2,3*^

^†^These authors contributed equally to this work.

^*^Corresponding author: Chenggang Wang

Tel./Fax. +86 0551-65786212

E-mail: cgwang@ahau.edu.cn

^1^College of Horticulture, Vegetable Genetics and Breeding Laboratory, Anhui Agricultural University, 130 West Changjiang Road, 230036 Hefei, Anhui, China;

^2^Provincial Engineering Laboratory for Horticultural Crop Breeding of Anhui, 130 West of Changjiang Road, 230036 Hefei, Anhui, China;

^3^Wanjiang Vegetable Industrial Technology Institute, Maanshan, Anhui, 238200, China

Table S4

DEGs of porphyrin and Chl metabolism pathways.

| Gene_ ID | Log_2_FC  LTA/LTB | Up  Down | Log_2_FC NTA/NTB | Up  Down | Description | gene_ symbol |
| --- | --- | --- | --- | --- | --- | --- |
| LOC103837134 | -1.288454784 | Down | 0.278751211 |  | glutamate--tRNA ligase, cytoplasmic | At5g26710 |
| LOC103866471 | 0.910452341 |  | 1.310650683 | Up | glutamate--tRNA ligase, cytoplasmic-like | At5g26710 |
| LOC103832916 | -0.535191896 | Down | -1.247076349 |  | chlorophyll synthase, chloroplastic-like | CHLG |
| LOC103841124 | -1.082486484 | Down | 0.331072228 |  | chlorophyll synthase, chloroplastic-like | CHLG |
| LOC103848555 | 1.349236936 | Up | 0.37248102 |  | chlorophyll synthase, chloroplastic-like | CHLG |
| LOC103848641 | -1.027557153 | Down | -0.424830141 |  | chlorophyll synthase, chloroplastic | CHLG |
| LOC103869777 | 2.373571247 | Up | 0.244624394 |  | chlorophyll synthase, chloroplastic-like | CHLG |
| LOC103856025 | -2.154501333 | Down | 0.208122605 |  | magnesium-chelatase subunit ChlH, chloroplastic | CHLH |
| LOC103856423 | -1.390670823 | Down | 0.195111316 |  | magnesium-chelatase subunit ChlI-1, chloroplastic | CHLI1 |
| LOC103860994 | -1.350420049 | Down | 0.064410829 |  | magnesium-chelatase subunit ChlI-1, chloroplastic-like | CHLI1 |
| LOC103862873 | -1.046677503 | Down | -0.750134814 |  | magnesium protoporphyrin IX methyltransferase, chloroplastic-like | CHLM |
| LOC103831923 | -1.090724053 | Down | 0.68012571 |  | geranylgeranyl diphosphate reductase, chloroplastic | CHLP |
| LOC103872768 | 5.342070183 | Up | -1.146172205 |  | chlorophyllase-1 | CLH1 |
| LOC103845044 | -1.308764509 | Down | 0.941079581 |  | cytochrome c oxidase assembly protein COX15 | COX15 |
| LOC103843696 | -1.145049298 | Down | 2.436439203 | Up | coproporphyrinogen-III oxidase 1, chloroplastic-like | CPX1 |
| LOC103830076 | -1.244283597 | Down | 0.732157382 |  | magnesium-protoporphyrin IX monomethyl ester [oxidative] cyclase, chloroplastic | CRD1 |
| LOC103841596 | -1.491399591 | Down | 0.925041498 |  | magnesium-protoporphyrin IX monomethyl ester [oxidative] cyclase, chloroplastic | CRD1 |
| LOC103845814 | -1.467020179 | Down | 0.066516006 |  | divinyl chlorophyllide a 8-vinyl-reductase, chloroplastic | DVR |
| LOC103842138 | -1.716790305 | Down | -0.567404147 |  | ferrochelatase-2, chloroplastic | FC2 |
| LOC103846758 | -0.503812804 | Down | 1.126637359 |  | frataxin, mitochondrial | FH |
| LOC103837478 | -0.564030503 | Down | 1.146527841 | Up | glutamate-1-semialdehyde 2,1-aminomutase, chloroplastic | GSA |
| LOC103843327 | 4.392753693 | Up | 0.268501298 |  | glutamyl-tRNA reductase 2, chloroplastic | HEMA2 |
| LOC103855770 | -0.538818431 | Down | 1.255913539 | Up | porphobilinogen deaminase, chloroplastic-like | HEMC |
| LOC103847911 | -1.180241404 | Down | -1.056978604 | Down | heme oxygenase 1, chloroplastic | HO1 |
| LOC103855594 | -1.054568303 | Down | 0.062624359 |  | chlorophyll(ide) b reductase NOL, chloroplastic | NOL |
| LOC103863639 | -1.047982103 | Down | -0.682278727 |  | probable chlorophyll(ide) b reductase NYC1, chloroplastic | NYC1 |
| LOC103861694 | 2.118675252 | Up | 2.022390999 | Up | protochlorophyllide reductase B, chloroplastic-like | PORB |
| LOC103867162 | 0.187432951 |  | 1.341166923 | Up | protochlorophyllide reductase B, chloroplastic | PORB |
| LOC103836607 | -1.860993828 | Down | -0.034246395 |  | protochlorophyllide reductase C, chloroplastic-like | PORC |
| LOC103844489 | -1.680122345 | Down | 0.459152089 |  | protochlorophyllide reductase C, chloroplastic | PORC |
| LOC103847701 | -2.028064484 | Down | -0.381369628 |  | pheophorbidase-like | PPD |
| LOC103869295 | -1.45781511 | Down | -1.931305965 | Down | pheophorbidase | PPD |
| LOC103834271 | -1.008703915 | Down | -0.166652582 |  | protein STAY-GREEN 1, chloroplastic-like | SGR1 |
| NYE1 | 2.0223805 | Up | 0.401983873 |  | protein STAY-GREEN 1, chloroplastic | SGR1 |
| LOC103858612 | 0.168588095 |  | 2.27181758 | Up | protein STAY-GREEN 2, chloroplastic-like | SGR2 |
| LOC103847714 | -3.351662551 | Down | -0.231604073 |  | protein STAY-GREEN LIKE, chloroplastic | SGRL |
| LOC103832890 | -1.064058154 | Down | -0.653802578 |  | sirohydrochlorin ferrochelatase, chloroplastic | SIRB |
