# Supplementary material for: Comparative transcriptome analysis reveals that chlorophyll metabolism contributes to leaf color changes in wucai (Brassica campestris L.) in response to cold
Source: BMC Plant Biol. 2021 Sep 28;21:438. doi: 10.1186/s12870-021-03218-9 (PMC8477495; doi:10.1186/s12870-021-03218-9)
Supplement: Supplementary file 5 — Additional file 5: Table S5. DEGs of carotenoid biosynthesis and abscisic acid biosynthesis pathways. [file 12870_2021_3218_MOESM5_ESM.docx]

**Comparative Transcriptome Analysis Reveals that Chlorophyll Metabolism Contributes to Leaf Color Changes in Wucai (*Brassica campestris* L.) in Response to Cold**

Lingyun Yuan ^1,2,3†^, Liting Zhang ^1,2†^, Ying Wu ^1,2^, Yushan Zheng ^1,2^, Libing Nie ^1,2^, Shengnan Zhang ^1,2^, Tian Lan ^1,2^, Yang Zhao ^1,2^, Shidong Zhu ^1,2,3^, Jinfeng Hou ^1,2,3^, Guohu Chen ^1,2,3^, Xiaoyan Tang ^1,2,3^ and Chenggang Wang ^1,2,3*^

^†^These authors contributed equally to this work.

^*^Corresponding author: Chenggang Wang

Tel./Fax. +86 0551-65786212

E-mail: cgwang@ahau.edu.cn

^1^College of Horticulture, Vegetable Genetics and Breeding Laboratory, Anhui Agricultural University, 130 West Changjiang Road, 230036 Hefei, Anhui, China;

^2^Provincial Engineering Laboratory for Horticultural Crop Breeding of Anhui, 130 West of Changjiang Road, 230036 Hefei, Anhui, China;

^3^Wanjiang Vegetable Industrial Technology Institute, Maanshan, Anhui, 238200, China

Table S5

DEGs of carotenoid biosynthesis and abscisic acid biosynthesis pathways.

| Gene_ ID | Log_2_FC  LTA/LTB | Up  Down | Log_2_FC  NTA/NTB | Up  Down | gene_ symbol | Description |
| --- | --- | --- | --- | --- | --- | --- |
| LOC103832798 | 3.926049892 | Up | 0.804548813 |  | xanthoxin dehydrogenase-like | ABA2 |
| LOC103871215 | 4.121151787 | Up | 0.728307982 |  | xanthoxin dehydrogenase | ABA2 |
| BCH | 0.167745076 | Down | 1.521979995 |  | beta-carotene 3-hydroxylase 1, chloroplastic | BETA-OHASE |
| LOC103863442 | 0.125389493 | Down | 2.371148699 | Up | beta-carotene 3-hydroxylase 1, chloroplastic | BETA-OHASE |
| LOC103843494 | 0.477113879 | Down | 0.735348508 |  | prolycopene isomerase, chloroplastic | CRTISO |
| LOC103857276 | 4.320463041 | Up | 0.450140223 |  | abscisic acid 8'-hydroxylase 1-like | CYP707A1 |
| LOC103861043 | 9.271749033 | Up | 1.995086589 |  | abscisic acid 8'-hydroxylase 1 | CYP707A1 |
| LOC103849374 | 0.276590834 | Down | 1.535779695 |  | abscisic acid 8'-hydroxylase 2 | CYP707A2 |
| LOC103827806 | 85.51707144 | Up | 0.542592888 |  | abscisic acid 8'-hydroxylase 3-like | CYP707A3 |
| LOC103839129 | 33.01789767 | Up | 0.690579511 |  | abscisic acid 8'-hydroxylase 3 | CYP707A3 |
| LOC103869474 | 0.713445842 |  | 7.701593097 | Up | abscisic acid 8'-hydroxylase 4 | CYP707A4 |
| LOC103833794 | 0.464981127 | Down | 0.417669643 | Down | protein LUTEIN DEFICIENT 5, chloroplastic | CYP97A3 |
| LOC103850280 | 0.409037314 | Down | 0.730599689 |  | carotene epsilon-monooxygenase, chloroplastic | CYP97C1 |
| LOC103843711 | 0.295830315 | Down | 0.539380577 |  | beta-carotene isomerase D27, chloroplastic | D27 |
| LOC103870482 | 0.290985711 | Down | 0.728905472 |  | lycopene beta cyclase, chloroplastic | LCY1 |
| LOC103845139 | 0.374310472 | Down | 1.156443956 |  | lycopene epsilon cyclase, chloroplastic-like | LUT2 |
| LOC103856778 | 0.215787447 | Down | 0.608955799 |  | lycopene epsilon cyclase, chloroplastic | LUT2 |
| LOC103870025 | 3.836109394 |  | 2.283154078 | Up | 9-cis-epoxycarotenoid dioxygenase NCED3, chloroplastic | NCED3 |
| LOC103835193 | 0.387980782 | Down | 0.573878079 |  | 15-cis-phytoene desaturase, chloroplastic/chromoplastic | PDS |
| LOC103846262 | 0.321942845 | Down | 0.656863688 |  | phytoene synthase, chloroplastic | PSY1 |
| LOC103851090 | 0.193633301 | Down | 0.645019102 |  | phytoene synthase, chloroplastic-like | PSY1 |
| LOC103856237 | 0.323991706 | Down | 0.689476973 |  | phytoene synthase, chloroplastic-like | PSY1 |
| LOC103871599 | 0.375178579 | Down | 0.828315746 |  | violaxanthin de-epoxidase, chloroplastic | VDE1 |
| LOC103849152 | 0.323818846 | Down | 0.549262691 |  | zeta-carotene desaturase, chloroplastic/chromoplastic | ZDS1 |
| LOC103837729 | 0.432120259 | Down | 0.771257755 |  | zeaxanthin epoxidase, chloroplastic-like | ZEP |
| LOC103871859 | 0.285003717 | Down | 0.985739541 |  | 15-cis-zeta-carotene isomerase, chloroplastic | Z-ISO |
