# Supplementary material for: Comparative transcriptome analysis reveals that chlorophyll metabolism contributes to leaf color changes in wucai (Brassica campestris L.) in response to cold
Source: BMC Plant Biol. 2021 Sep 28;21:438. doi: 10.1186/s12870-021-03218-9 (PMC8477495; doi:10.1186/s12870-021-03218-9)
Supplement: Supplementary file 6 — Additional file 6: Table S6. DEGs of photosynthesis and the photosynthesis-antenna pathway. [file 12870_2021_3218_MOESM6_ESM.docx]

**Comparative Transcriptome Analysis Reveals that Chlorophyll Metabolism Contributes to Leaf Color Changes in Wucai (*Brassica campestris* L.) in Response to Cold**

Lingyun Yuan ^1,2,3†^, Liting Zhang ^1,2†^, Ying Wu ^1,2^, Yushan Zheng ^1,2^, Libing Nie ^1,2^, Shengnan Zhang ^1,2^, Tian Lan ^1,2^, Yang Zhao ^1,2^, Shidong Zhu ^1,2,3^, Jinfeng Hou ^1,2,3^, Guohu Chen ^1,2,3^, Xiaoyan Tang ^1,2,3^ and Chenggang Wang ^1,2,3*^

^†^These authors contributed equally to this work.

^*^Corresponding author: Chenggang Wang

Tel./Fax. +86 0551-65786212

E-mail: cgwang@ahau.edu.cn

^1^College of Horticulture, Vegetable Genetics and Breeding Laboratory, Anhui Agricultural University, 130 West Changjiang Road, 230036 Hefei, Anhui, China;

^2^Provincial Engineering Laboratory for Horticultural Crop Breeding of Anhui, 130 West of Changjiang Road, 230036 Hefei, Anhui, China;

^3^Wanjiang Vegetable Industrial Technology Institute, Maanshan, Anhui, 238200, China

c Table S6

DEGs of photosynthesis and the photosynthesis-antenna pathway.

| Gene_ ID | Log_2_FC | | Up | | | Log_2_FC | | Up | | Description | gene_ symbol |
| --- | --- | --- | --- | --- | --- | --- | --- | --- | --- | --- | --- |
|  | LTA/LTB | | Down | | | NTA/NTB | | Down | |  |  |
| Photosynthesis - antenna proteins | | | | |  | |  | |  | |  |
|  |  |  |  |  |  | |  | |  | |  |
| LOC103857533 | | 0.130470055 | |  | -1.230418691 | | Down | | chlorophyll a-b binding protein 1, chloroplastic | | CAB1 |
| LOC103865334 | | -0.011312054 | |  | -1.824726028 | | Down | | chlorophyll a-b binding protein 1, chloroplastic-like | | CAB1 |
| LOC103867454 | | 1.137388859 | | Up | -4.002403925 | | Down | | chlorophyll a-b binding protein 1, chloroplastic-like | | CAB1 |
| LOC103867457 | | 0.592099547 | |  | -1.399772913 | | Down | | chlorophyll a-b binding protein 1, chloroplastic-like | | CAB1 |
| LOC103842881 | | 0.358064403 | |  | -1.730729057 | | Down | | chlorophyll a-b binding protein CP24, chloroplastic-like | | CAP10B |
| LOC103829997 | | -0.588749331 | |  | -1.560668598 | | Down | | chlorophyll a-b binding protein 6, chloroplastic | | LHCA1 |
| LOC103841392 | | -0.08616677 | |  | -3.09175646 | | Down | | chlorophyll a-b binding protein 6, chloroplastic | | LHCA1 |
| LOC103873241 | | 0.171202177 | |  | -1.574758773 | | Down | | chlorophyll a-b binding protein 4, chloroplastic | | LHCA4 |
| LOC103828916 | | -0.766458242 | |  | -2.170925302 | | Down | | chlorophyll a-b binding protein 1, chloroplastic | | LHCB1.3 |
| LOC103828920 | | -1.356722749 | | Down | -1.746280952 | | Down | | chlorophyll a-b binding protein 1, chloroplastic-like | | LHCB1.3 |
| LOC103860327 | | -0.355692037 | |  | -1.621980769 | | Down | | chlorophyll a-b binding protein 151, chloroplastic | | LHCB2.1 |
| LOC103844887 | | 0.195816649 | |  | -2.370164858 | | Down | | chlorophyll a-b binding protein 3, chloroplastic | | LHCB3 |
| LOC103850412 | | -0.09630889 | |  | -1.165161394 | | Down | | chlorophyll a-b binding protein CP29.1, chloroplastic | | LHCB4.1 |
| LOC103870577 | | 1.171738021 | | Up | -1.537294835 | | Down | | chlorophyll a-b binding protein CP29.2, chloroplastic | | LHCB4.2 |
| LOC103866983 | | -1.317166998 | | Down | 0.202992581 | |  | | chlorophyll a-b binding protein CP29.3, chloroplastic | | LHCB4.3 |
| LOC103838910 | | -0.64024796 | |  | -1.227070627 | | Down | | chlorophyll a-b binding protein CP26, chloroplastic-like | | LHCB5 |
| Photosynthesis | | | | |  | |  | |  | |  |
|  |  |  |  |  |  | |  | |  | |  |
| LOC103858708 | | -1.102385364 | | Down | -1.047064434 | | Down | | cytochrome b6-f complex iron-sulfur subunit, chloroplastic | | petC |
| LOC103832056 | | -1.103387397 | | Down | 0.879881138 | |  | | plastocyanin | | PETE |
| LOC103842995 | | -1.239124839 | | Down | -0.880018483 | |  | | photosynthetic NDH subunit of lumenal location 2, chloroplastic | | PNSL2 |
| LOC103872111 | | -1.46385953 | | Down | -0.419833588 | |  | | photosynthetic NDH subunit of lumenal location 2, chloroplastic-like | | PNSL2 |
| LOC103850067 | | -1.980652081 | | Down | -0.794752062 | |  | | photosynthetic NDH subunit of lumenal location 3, chloroplastic-like | | PNSL3 |
| LOC103870981 | | -2.136991984 | | Down | -0.414971971 | |  | | photosynthetic NDH subunit of lumenal location 3, chloroplastic | | PNSL3 |
| LOC103863173 | | -1.184336721 | | Down | -0.90192527 | |  | | psbP-like protein 1, chloroplastic | | PPL1 |
| LOC103828782 | | -0.48806659 | |  | -1.290565254 | | Down | | photosystem I reaction center subunit III, chloroplastic-like | | PSAF |
| LOC103868386 | | -1.018226686 | | Down | -0.929369234 | |  | | photosystem I reaction center subunit V, chloroplastic | | PSAG |
| LOC103855224 | | -0.130173323 | |  | -1.452488832 | | Down | | photosystem I reaction center subunit N, chloroplastic-like | | PSAN |
| LOC103836610 | | -1.709502032 | | Down | -0.305535559 | |  | | photosystem II repair protein PSB27-H1, chloroplastic-like | | PSB27-1 |
| LOC103854241 | | -2.216976666 | | Down | 0.472509956 | |  | | photosystem II reaction center PSB28 protein, chloroplastic | | PSB28 |
| LOC103861848 | | -1.812957197 | | Down | -0.042536913 | |  | | photosystem II reaction center PSB28 protein, chloroplastic-like | | PSB28 |
| LOC103837695 | | -1.029300808 | | Down | -0.476547998 | |  | | oxygen-evolving enhancer protein 1-1, chloroplastic | | PSBO1 |
| LOC103843499 | | -1.23913397 | | Down | -0.238919116 | |  | | oxygen-evolving enhancer protein 2, chloroplastic | | PSBP |
| LOC103867933 | | -1.100329478 | | Down | -0.305925435 | |  | | oxygen-evolving enhancer protein 2-1, chloroplastic-like | | PSBP1 |
| LOC103858917 | | -1.088457088 | | Down | 0.270257546 | |  | | oxygen-evolving enhancer protein 3-1, chloroplastic | | PSBQ2 |
| LOC103832382 | | -1.094018829 | | Down | -0.724559062 | |  | | photosystem II 10 kDa polypeptide, chloroplastic-like | | PSBR |
| LOC103833131 | | -1.764687856 | | Down | -0.491094278 | |  | | photosystem II 22 kDa protein, chloroplastic | | PSBS |
| LOC103847722 | | -1.039736016 | | Down | -0.209228385 | |  | | photosystem II 22 kDa protein, chloroplastic | | PSBS |
| LOC103831013 | | -1.68161403 | | Down | -0.037764148 | |  | | photosystem II core complex proteins psbY, chloroplastic-like | | PSBY |
| LOC103831199 | | -1.978921008 | | Down | 0.161103183 | |  | | photosystem II core complex proteins psbY, chloroplastic | | PSBY |
| LOC103852422 | | -1.451304296 | | Down | -0.261432368 | |  | | photosystem II core complex proteins psbY, chloroplastic-like | | PSBY |
| LOC103839748 | | 1.03715482 | | Up | 1.413581747 | | Up | | ferredoxin--NADP reductase, root isozyme 1, chloroplastic | | RFNR1 |
| LOC103840388 | | 1.061697971 | | Up | -0.428057838 | |  | | ferredoxin--NADP reductase, root isozyme 2, chloroplastic | | RFNR2 |
| LOC103839821 | | -1.243148455 | | Down | -0.231115775 | |  | | ATP synthase gamma chain 1, chloroplastic-like | | ATPC1 |
| LOC103858669 | | -1.268789305 | | Down | -0.359884351 | |  | | ATP synthase gamma chain 1, chloroplastic | | ATPC1 |
| LOC103868164 | | -2.615208752 | | Down | 0.666674502 | |  | | ATP synthase gamma chain 1, chloroplastic-like | | ATPC1 |
| LOC103838839 | | -1.42188083 | | Down | -0.097118484 | |  | | ATP synthase subunit delta, chloroplastic | | ATPD |
| LOC103858533 | | -1.649397391 | | Down | -0.271679202 | |  | | ATP synthase subunit delta, chloroplastic-like | | ATPD |
| LOC103851204 | | -1.246726822 | | Down | -0.319575388 | |  | | ATP synthase subunit b', chloroplastic | | ATPG |
