# Supplementary material for: Comparative transcriptome analysis reveals that chlorophyll metabolism contributes to leaf color changes in wucai (Brassica campestris L.) in response to cold
Source: BMC Plant Biol. 2021 Sep 28;21:438. doi: 10.1186/s12870-021-03218-9 (PMC8477495; doi:10.1186/s12870-021-03218-9)
Supplement: Supplementary file 7 — Additional file 7: Table S7. DEGs of the circadian rhythm pathway. [file 12870_2021_3218_MOESM7_ESM.docx]

**Comparative Transcriptome Analysis Reveals that Chlorophyll Metabolism Contributes to Leaf Color Changes in Wucai (*Brassica campestris* L.) in Response to Cold**

Lingyun Yuan ^1,2,3†^, Liting Zhang ^1,2†^, Ying Wu ^1,2^, Yushan Zheng ^1,2^, Libing Nie ^1,2^, Shengnan Zhang ^1,2^, Tian Lan ^1,2^, Yang Zhao ^1,2^, Shidong Zhu ^1,2,3^, Jinfeng Hou ^1,2,3^, Guohu Chen ^1,2,3^, Xiaoyan Tang ^1,2,3^ and Chenggang Wang ^1,2,3*^

^†^These authors contributed equally to this work.

^*^Corresponding author: Chenggang Wang

Tel./Fax. +86 0551-65786212

E-mail: cgwang@ahau.edu.cn

^1^College of Horticulture, Vegetable Genetics and Breeding Laboratory, Anhui Agricultural University, 130 West Changjiang Road, 230036 Hefei, Anhui, China;

^2^Provincial Engineering Laboratory for Horticultural Crop Breeding of Anhui, 130 West of Changjiang Road, 230036 Hefei, Anhui, China;

^3^Wanjiang Vegetable Industrial Technology Institute, Maanshan, Anhui, 238200, China

Table S7

DEGs of the circadian rhythm pathway.

| Gene_ ID | Log_2_FC | Up | Log_2_FC | Up | Description | gene_ symbol | |
| --- | --- | --- | --- | --- | --- | --- | --- |
|  | LTA/LTB | Down | NTA/NTB | Down |  |  |  |
| LOC103837378 | 0.438520439 |  | 1.836113804 | Up | two-component response regulator-like APRR1 | | APRR1 |
| LOC103860589 | -1.398689847 | Down | -0.237509877 |  | two-component response regulator-like APRR1 | | APRR1 |
| LOC103845423 | 3.4656784 | Up | 0.679796624 |  | two-component response regulator-like APRR3 | | APRR3 |
| LOC103837062 | -2.702107746 | Down | 0.595044158 |  | two-component response regulator-like APRR5 | | APRR5 |
| LOC103854859 | -2.621001744 | Down | 1.369724182 | Up | two-component response regulator-like APRR5 | | APRR5 |
| LOC103874318 | -1.984962575 | Down | 2.28217802 | Up | two-component response regulator-like APRR5 | | APRR5 |
| LOC103866306 | -4.301237395 | Down | -0.544966104 |  | two-component response regulator-like APRR9 | | APRR9 |
| LOC103866432 | -4.115060784 | Down | 0.416633716 |  | two-component response regulator-like APRR9 | | APRR9 |
| LOC103855006 | -2.4447372 | Down | 2.192550869 | Up | cyclic dof factor 1-like | | CDF1 |
| LOC103874677 | -3.180107703 | Down | 0.901464733 |  | cyclic dof factor 1 | | CDF1 |
| LOC103846527 | -2.900294545 | Down | -0.508128497 |  | chalcone synthase 1 | | CHS1 |
| LOC103850907 | -2.233095093 | Down | -0.202122649 |  | chalcone synthase 3-like | | CHS3 |
| LOC103856040 | -2.498025306 | Down | -0.676049692 |  | chalcone synthase 3-like | | CHS3 |
| LOC103855586 | 1.116642381 | Up | -0.347780942 |  | casein kinase II subunit beta'-like | | CKB2 |
| LOC103844260 | -1.235905004 | Down | 0.081920893 |  | cryptochrome-2 | | CRY2 |
| HY5 | -1.100766276 | Down | -0.63281787 |  | transcription factor HY5 | | HY5 |
| LOC103850763 | -2.779812473 | Down | -0.771330601 |  | transcription factor HY5-like | | HY5 |
| LOC103839184 | -1.430814618 | Down | -1.328087801 |  | transcription factor HY5-like | | HYH |
| LOC103859717 | -3.12295098 | Down | 0.680736088 |  | transcription factor HY5-like | | HYH |
| LOC103869673 | -3.588242024 | Down | 0.628843635 |  | transcription factor HY5-like | | HYH |
| LOC103836661 | -1.391660456 | Down | -2.156677098 | Down | protein LHY-like | | LHY |
| LOC103844616 | -1.548908465 | Down | -2.533567893 | Down | protein LHY | | LHY |
| LOC103871728 | 1.281169512 | Up | 0.438409593 |  | phytochrome A | | PHYA |
| LOC103871725 | 2.490530735 | Up | 1.20069633 | Up | transcription factor PIF3 | | PIF3 |
| LOC103843349 | 2.399619359 | Up | 0.214561306 |  | transcription factor PIF3-like | | PIF3 |
| LOC103865631 | -3.826792709 | Down | -2.28660909 |  | WD repeat-containing protein RUP2 | | RUP2 |
| LOC103858211 | -1.917825758 | Down | 0.910531682 |  | protein SUPPRESSOR OF PHYA-105 1-like | | SPA1 |
| LOC103847150 | 1.405079184 | Up | -0.673601046 |  | transcription factor TCP21-like | | TCP21 |
| LOC103850694 | 3.221039347 | Up | 0.341533217 |  | transcription factor TCP21-like | | TCP21 |
| LOC103855775 | 2.962873877 | Up | -0.116948756 |  | transcription factor TCP21 | | TCP21 |
| LOC103865427 | 1.340401972 | Up | 2.178300583 | Up | transcription factor TCP7-like | | TCP7 |
| LOC103874198 | 1.876796871 | Up | 0.546764522 |  | transcription factor TCP7-like | | TCP7 |
